# An efficient YOLOv12-based framework for detecting extremely small-scale objects

**DOI:** 10.1038/s41598-025-31803-7

**Published:** 2025-12-12

**Authors:** A. Chandrashekhar, B. Satyanarayana, Rajani Reddy Gorrepati, Ponduri Vasanthi, Kothala Lakshmi Prasanna

**Affiliations:** 1 Department of Mechanical Engineering, Faculty of Science and Technology, Icfai Foundation for Higher Education, Hyderabad, 501203 Telangana India; 2 Department of ECE, SASI institute of Technology and Engineering, Tadepalligudem, India; 3 Department of Computer Science and Engineering, Koneru Lakshmaiah Education Foundation, Vaddeswaram, Andhra Pradesh India; 4 Department of Electronics and Communication Engineering, Eswar College of Engineering, Narasaraopet, Guntur, 522601 Andhra Pradesh India; 5 Department of Electronics and Communication Engineering, ACE Engineering College, Hyderabad, India

**Keywords:** Area-Attention C2f, C3K2, Multi-scale feature fusion, Extremely small object detection, Lightweight attention module, Decoupled detection head, Engineering, Mathematics and computing

## Abstract

Object detection in aerial imagery, particularly from UAVs and drones, presents unique challenges such as small and extremely small-scale object distributions. To address these drawbacks, the present research proposes an efficient YOLOv12 model. The first core contribution of our work is the introduction of the Area-Attention C2f (A2C2F) module, which fuses multi-head MLP blocks with localized area-attention mechanisms to strengthen spatial feature learning while maintaining lightweight computation. Second, the model integrates the Cross Stage Partial with Kernel size 2 (C3K2) module, a lightweight variant of the C3 block, which reduces convolutional complexity and computational cost while maintaining feature extraction capability for efficient detection. Third, a multi-scale fusion strategy by stacking A2C2F blocks along with Concat and Upsample operations ensures the preservation of high-resolution features for detecting small-scale objects, a common challenge in drone-captured data. Fourth, a novel detection head with decoupled classification and regression branches, enhanced by attention-guided feature fusion, improves prediction consistency and boosts inference speed. Extensive experiments conducted on the VisDrone dataset demonstrate that the proposed framework effectively detects objects with dimensions smaller than 3–5 pixels, where traditional YOLO variants typically fail, proving its robustness in extremely small-scale detection. The proposed model achieves a Precision of 69.1%, Recall of 48.5%, and F1-score of 56.99%, outperforming recent models. Notably, it achieves a mAP@50 of 58.8% and mAP@0.5:0.95 of 40.9%, showing substantial improvement over prior methods. Despite utilizing 59.1 M parameters and 198.6 GFLOPs, the model maintains an efficient inference rate of approximately 40 FPS on the A100 GPU, confirming its suitability for real-time UAV edge deployment. When compared to two-stage and anchor-based models, our model delivers higher AP scores at a lower input resolution of 640 × 640 pixels, making it a highly effective and computationally efficient framework for UAV-based small and extremely small object detection.

**Supplementary Information:**

The online version contains supplementary material available at 10.1038/s41598-025-31803-7.

## Introduction

Small object detection in aerial imagery, particularly from unmanned aerial vehicles (UAVs), has emerged as a critical challenge in the computer vision community due to the proliferation of drone-based applications in agriculture, surveillance, environmental monitoring, and disaster management. Despite advancements in deep learning-based object detectors such as the YOLO and R-CNN families, detecting objects that occupy a minimal number of pixels within high-resolution aerial images remains a persistent problem. The primary issues lie in the inadequate semantic representation of small objects, the imbalance of scale variation, and the dilution of feature granularity during deep convolutional operations. Traditional object detection frameworks often fail to capture fine-grained spatial features necessary for identifying small-scale targets. This deficiency necessitates the development of specialized detection mechanisms that fuse multi-scale features, enhance semantic content, and leverage attention mechanisms to amplify discriminative regions. Moreover, real-time detection capability and deployment feasibility on resource-constrained UAV platforms demand lightweight yet highly accurate solutions. In recent years, the integration of shallow feature fusion techniques, transformer modules, anchor-free approaches, and attention-guided mechanisms has significantly improved detection performance for small objects in aerial settings. These techniques aim to maintain contextual integrity while ensuring computational efficiency, opening new possibilities for reliable and scalable detection models suitable for UAV-based applications.

Detecting extremely small-scale objects (often smaller than 3 pixels) in aerial imagery presents several unique challenges. Such objects occupy very limited pixel regions, making easily indistinguishable their appearance features such as shape, texture, and edges. Moreover, due to strong scale variation and complex backgrounds, these tiny objects are easily lost during downsampling in deep convolutional networks. The imbalance between small targets and large background areas further reduces detection sensitivity. These difficulties motivated the design of the proposed framework, where the C3K2 backbone preserves fine spatial details through compact kernel convolution, and the A2C2F module introduces area-guided attention to amplify weak object cues while suppressing background noise. Together, these components enable more accurate localization and classification of extremely small targets in UAV-based aerial scenes.

## Literature survey

With the proliferation of unmanned aerial vehicles (UAVs), object detection in drone-captured imagery has gained significant attention. Luo et al. (2021) introduced a method that combines shallow feature fusion with semantic information enhancement to improve object detection accuracy, particularly benefiting small object representation by preserving low-level spatial features^1^. Similarly, Song et al. (2022) proposed MSF-YOLO, which utilizes a multi-scale feature fusion strategy to strengthen the network’s focus on small targets, thereby enhancing performance in cluttered backgrounds^2^. Jin et al. (2021) tackled the challenge of scale variation by presenting an extreme-scale metric learning approach tailored for aerial images, which boosts intra-class compactness and inter-class separability to better distinguish small objects^3^. Zhan et al. (2022)optimized YOLOv5 for UAV-based real-time detection, incorporating a refined loss function and custom anchor design, achieving notable accuracy gains for small objects^4–6^. Law and Deng (2018) proposed CornerNet, which detects object corners instead of bounding boxes, offering an alternative for precise localization of small targets^7^. Li et al. (2017) developed Light-Head R-CNN to reduce the computational load of two-stage detectors while maintaining accuracy, which is crucial for real-time UAV applications^8^.

Lin et al. (2017) introduced Feature Pyramid Networks (FPN), a landmark approach that exploits multi-scale feature representations to improve detection across varying object sizes^9^. Building upon this, Wan et al. (2021) presented ViStrongerDet for detecting dense and small objects in VisDrone images by strengthening the visual feature extraction capacity^10^. Recent innovations focus heavily on integrating attention mechanisms and transformer architectures. Vasanthi and Mohan (2024) proposed a Multi-Head Self-Attention based YOLOv5X-transformer model, significantly improving detection precision for multi-scale objects, including extremely small ones^11^. Their earlier work (2023) introduced an anchor-regenerative transformer model targeting x-small and dense objects, demonstrating enhanced feature localization in high-clutter UAV imagery^12^. Kothala et al. (2023) employed ghost convolutions in a YOLO-based framework for medical image localization, demonstrating the lightweight module’s potential for small object detection^13^. Their subsequent model, TL-LFF Net, employed transfer learning and genetic optimization to achieve faster and more accurate small-object recognition^14^.

Yue et al. (2024) enhanced YOLOv8n to create a lightweight yet efficient detector tailored for UAV images, optimizing it for real-time applications on limited hardware^15^. Jiang et al. (2024) designed MFFSODNet, which utilizes multi-scale feature fusion to increase the representation power for detecting small UAV targets^16^. Su et al. (2024) proposed MPE-YOLO, emphasizing enhanced small-target detection in aerial imaging by refining feature propagation and anchor generation^17^. Similarly, Tang et al. (2024) developed HRYNet, a robust YOLO-based framework for detecting complex road traffic objects from UAV views^18^. Shi and Zhang (2024) introduced FocusDet, which emphasizes efficient feature extraction for small objects while reducing redundancy^19^. Li et al. (2024) proposed TA-YOLO, a model leveraging small object detection in remote sensing images, achieving better feature discrimination^20^. Zhang et al. (2024) presented APNet, which integrates deformable convolutions for more accurate spatial positioning of small UAV objects^21^.

Wu et al. (2024) introduced EUAVDet, an edge-efficient lightweight detector tailored for aerial imagery, supporting real-time processing with minimal computational demand^22^. Zhou et al. (2024) designed MFEFNet, focusing on extracting and fusing multi-scale features to enhance detection under varied object scales and densities^23^. Huang et al. (2024) drew inspiration from biological vision systems in their BRSTD model, aiming for more adaptive detection in remote sensing images^24^. Li et al. (2024) developed SOD-YOLO, an improved version of YOLOv8 that focuses on better bounding box regression for small UAV objects^25^. Wang et al. (2024) proposed AMFEF-DETR, an end-to-end network that integrates adaptive multi-scale feature extraction and fusion specifically for UAV data^26^. Tan et al. (2024) also highlighted adaptive fusion mechanisms in their model, which adjusts feature emphasis based on object scale^27^.

Jing et al. (2024) proposed MVT, a multi-vision transformer designed for event-based small target detection, introducing dynamic spatial processing in aerial imagery^28^. Zhang et al. (2024) introduced HSP-YOLOv8, specifically optimized for high-speed UAV aerial photography involving tiny target detection^29^. Vasanthi and Mohan (2024) further improved YOLOv8 by developing a highly efficient version for extreme small-scale object detection, achieving superior accuracy with reduced model size^30^. Finally, Ponduri et al. (2025) introduced a novel YOLOv9 model designed for extremely small-scale objects, demonstrating significant improvements in both speed and detection accuracy^31^. Additionally, Tang et al. (2024) developed LCFF-Net, a lightweight cross-scale feature fusion network that enhances the detection of tiny targets in UAV imagery by integrating shallow and deep features more effectively^32^.

Li et al. (2022) proposed an anchor-free lightweight deep convolutional network for vehicle detection in aerial imagery, demonstrating that anchor-free designs can effectively reduce computation while maintaining detection precision^34^. Wang et al. (2024) introduced a hybrid attention-based lightweight CNN for fine-grained instrument indication recognition, highlighting the role of hybrid attention in improving small target discrimination^35^. Zhang et al. (2025) presented a lightweight semantic feature extraction network for ancient mural object detection, showcasing efficient feature reuse strategies applicable to fine-grained texture preservation in small objects^36^. Chen et al. (2022) proposed a lightweight CNN-based finger vein recognition model, which achieved high performance with minimal parameters, illustrating the effectiveness of compact network designs for high-precision applications^37^. These works collectively emphasize the increasing research focus on lightweight architectures that balance accuracy and efficiency. Later, Zhao et al.^38^ proposed a dynamic feature integration framework that emphasizes selective and adaptive feature fusion, which inspired the selective feature refinement process used in our multi-scale fusion design. Luo et al.^39^ introduced multi-level contextual aggregation strategies that influenced our approach to preserving high-resolution spatial information through stacked A2C2F blocks. Similarly, Sun et al.^40^ explored attention mechanism linearization and diffusion-based learning, motivating our emphasis on efficient and localized attention modeling within the A2C2F module. Furthermore, Chen et al.^41^ investigated architecture optimization for lightweight detection, which aligns with our design of the C3K2 lightweight convolutional module aimed at reducing computational cost while maintaining detection accuracy. Specifically, Liu et al. (47), employed wavelet pooling and graph-enhanced classification to address small-object tracking challenges in UAV imagery. While this approach effectively enhances contextual awareness, it primarily targets object tracking rather than detection and incurs higher computational overhead due to graph-based processing.

Wang et al. (2025) introduces a frequency-aware interaction and multi-expert fusion framework that enhances robustness under complex illumination and motion conditions, demonstrating the potential of adaptive frequency feature learning for UAV-based tracking tasks^43^. Li et al. (2025), employs a dynamic sparse learning mechanism to facilitate aerial vision-language tracking, thereby enabling semantically guided UAV perception through efficient cross-modal representation^44^. Furthermore, Zhang et al. (2024) proposed the recent work on query-guided redemption presents an occlusion-resilient UAV visual tracking approach, effectively mitigating object loss in challenging aerial scenes^45^. In addition, Chen et al. (2025) proposes a global agent framework to improve discrimination between targets and distractors, contributing to stable and high-precision tracking performance^46^. In contrast, our proposed A2C2F and C3K2 modules are designed for efficient small-object detection, offering superior lightweight computation, faster inference, and localized spatial feature enhancement without relying on complex graph representations.

The primary motivation of the proposed model is to address the difficulty of detecting extremely small-scale objects, which are often missed by conventional YOLO-based detectors due to loss of fine spatial information and weak multi-scale fusion. Existing models struggle to preserve local detail and contextual balance at sub-pixel levels. Hence, the proposed model introduces localized area-attention and adaptive fusion modules to enhance fine-grained feature preservation, improve sensitivity to tiny targets, and achieve accurate detection under extreme small-scale conditions.

The literature section now clearly synthesizes the following limitations:

1. Loss of fine spatial details: Many YOLO-based and multi-scale fusion methods struggle to retain sub-pixel level information, causing frequent mis-detections of extremely small objects.
2. Insufficient localized context modeling: Transformer-based and attention-driven methods often emphasize global context, but lack efficient localized spatial refinement necessary for tiny target representation.
3. High computational overhead: Several recent transformer and graph-based approaches deliver accuracy improvements but introduce significant complexity, making them unsuitable for lightweight UAV deployment.
4. Weak cross-scale feature interaction: Existing feature fusion mechanisms may underutilize shallow features, leading to imbalanced contextual representation for small object detection.
5. Limited real-time efficiency: Methods based on heavy backbones or multi-stage processing fail to meet the strict real-time constraints of UAV platforms.

These limitations motivated the design of our A2C2F and C3K2 modules. The revised text highlights that our model specifically targets localized detail preservation, adaptive multi-scale fusion, and lightweight computation to address gaps in prior work. These additions improve the logical flow of Sect. 2 by connecting the reviewed literature directly to the motivations and innovations of our proposed approach.

The contributions of the proposed work can be written as,

1. Enhanced Spatial Representation: Introduction of the Area-Attention C2f (A2C2F) module, which integrates area-based attention with MLP blocks to strengthen spatial feature learning and capture localized context efficiently.
2. Lightweight Feature Extraction: Integration of the C3K2 module, a computationally efficient variant of the C3 block, designed to minimize convolutional complexity and parameter count while maintaining rich feature extraction capability.
3. Multi-Scale and Adaptive Detection: Development of an optimized detection pipeline combining multi-scale fusion (Concat + Upsample + stacked A2C2F) and an attention-guided multi-scale detection head, improving precision and inference speed for extremely small-scale objects.

The total organization of the paper is as follows: Sect. 1 gives the introduction of extreme small-scale objects along with the motivation of the proposed method. Section 2 gives a detailed literature survey of VisDrone data based on traditional and YOLO based models. Section 3 describes the proposed YOLOv12 based enhanced detection framework, including its overall architecture and key components. Section 4 details the experimental setup, dataset characteristics, evaluation metrics, and implementation strategy. It also discusses the results, highlighting the model’s performance in comparison with existing methods. Finally, the conclusion, limitations, are also discussed.

## Proposed model

**Fig. 1:**
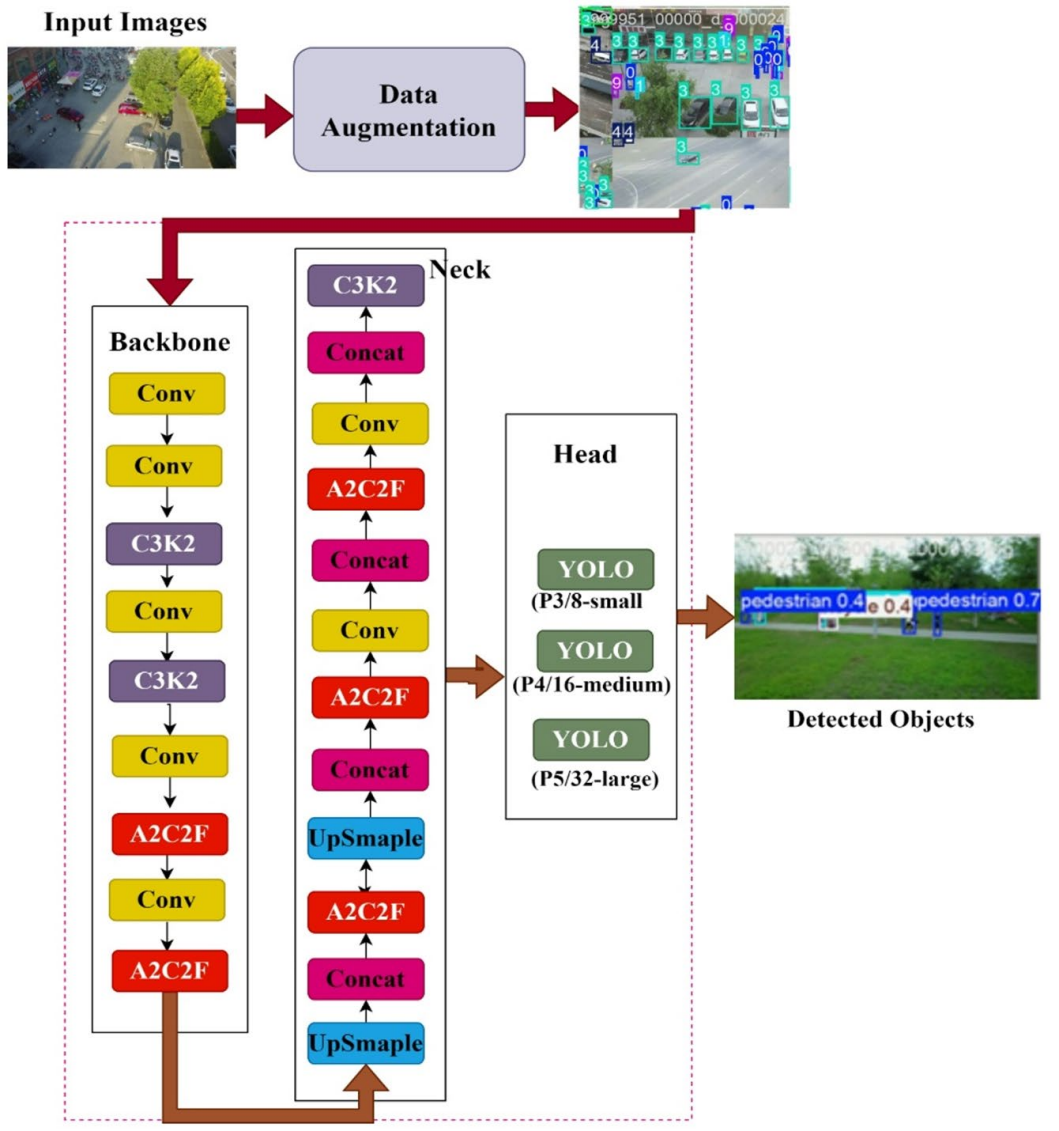
Layer Architecture of the proposed model.

In this work, an efficient YOLOv12-based object detection architecture tailored for small-scale object detection scenarios, such as pedestrian and vehicle detection in aerial surveillance imagery. The architecture key innovations in the backbone (C3K2), attention fusion modules (A2C2F), and multi-scale feature aggregation and multi scale adaptive detection heads are shown in Fig. 1. These modifications improve feature representation, spatial resolution preservation, and contextual awareness.

### Data augmentation

A robust data augmentation strategy is employed to enhance the generalization capability of the object detection model, particularly in scenarios involving limited data availability or class imbalance. The augmentation process integrates three key techniques: Mosaic Augmentation, HSV color space adjustments, and Random Cropping and Scaling. Mosaic Augmentation improves context learning and object scale variation handling by combining four different images into a single training image through random scaling and quadrant-wise concatenation. HSV adjustment simulates environmental lighting variations by randomly modifying the hue, saturation, and brightness values of the image in HSV color space. Random Cropping and Scaling further improves robustness by training the model to recognize partially visible and spatially shifted objects, achieved by selecting a random subregion of the image and resizing it to the original input size.

### Backbone feature extractor

Each Conv block applies a linear transformation followed by batch normalization and a non-linearity:

1 $$\:\mathrm{Y}={\upsigma\:}\left(\mathrm{B}\mathrm{N}\left(\mathrm{W}\mathrm{*}\mathrm{X}+\mathrm{b}\right)\right)$$

where ∗ denotes convolution, and σ is a non-linear activation function.

### C3K2 module

The C3K2 module is introduced as a lightweight variant of the standard YOLOv8 C3 block. Traditional convolutional backbones achieve high accuracy but involve significant convolutional complexity and redundant feature extraction, which increase inference latency. The C3K2 module modifies the kernel configuration by employing a kernel size of 2 × 2 instead of the conventional 3 × 3, thereby reducing the number of parameters and FLOPs without sacrificing representational capacity. This design choice achieves a favorable balance between computational efficiency and feature richness, enabling faster inference while retaining strong performance on small and extremely small object categories. In the C3K2 module, the input X is split into two parts:

2 $$\:{\mathrm{X}}_{1},{\mathrm{X}}_{2}=\mathrm{s}\mathrm{p}\mathrm{l}\mathrm{i}\mathrm{t}\:\left(\mathrm{X}\right)$$

The second part is processed through multiple 2 × 2 convolutions with residual connections:

3 $$\:{\mathrm{Y}}_{1}={\mathrm{C}\mathrm{o}\mathrm{n}\mathrm{v}}_{2\times\:2}\left({\mathrm{X}}_{2}\right)$$

4 $$\:{\mathrm{Y}}_{2}={\mathrm{C}\mathrm{o}\mathrm{n}\mathrm{v}}_{2\times\:2}\left({\mathrm{Y}}_{1}\right)$$

Output is obtained via feature fusion:

5 $$\:\mathrm{Y}={\mathrm{C}\mathrm{o}\mathrm{n}\mathrm{v}}_{1\times\:1}\left(\mathrm{C}\mathrm{o}\mathrm{n}\mathrm{c}\mathrm{a}\mathrm{t}\left({\mathrm{X}}_{1},\:{\mathrm{Y}}_{2}\right)\right)$$

The C3K2 structure improves feature compactness while maintaining gradient flow across deeper layers.

### Area attention cross-connected fusion (A2C2F)

**Fig. 2:**
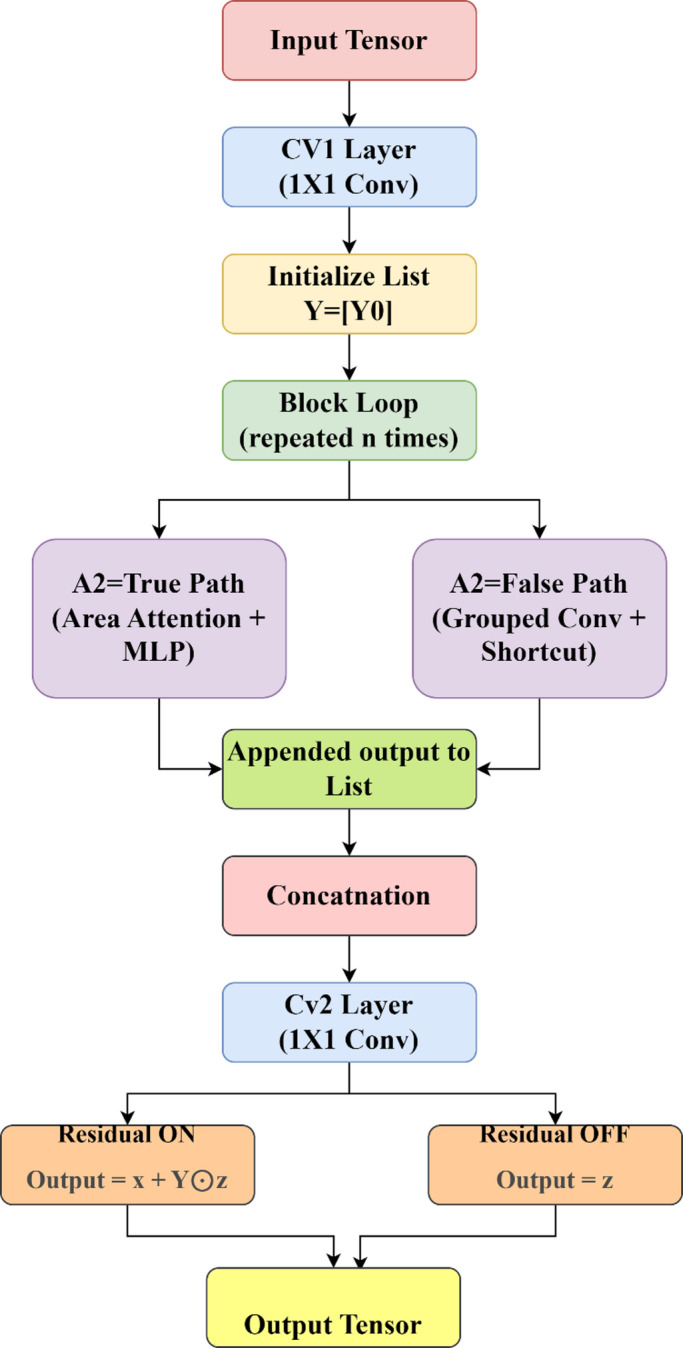
A2C2F module.

Traditional FPNs or PANets lack the dynamic ability to weigh features based on spatial and channel importance. The A2C2F (Area-Attention C2f) module was designed to overcome the limitations of conventional channel and spatial attention mechanisms (e.g., CBAM, SE, and ECA), which primarily focus on global or channel-level weighting and often fail to capture the localized context crucial for extremely small-scale object detection in UAV imagery. The proposed A2C2F integrates localized area-based attention with multi-head MLP blocks, allowing the model to emphasize fine-grained regional dependencies and spatial boundaries with minimal computational overhead. Unlike transformer-style global self-attention, which incurs high FLOPs and may dilute spatial detail, the A2C2F efficiently enhances local spatial coherence and context-aware representation while maintaining lightweight computation suitable for real-time aerial detection. The A2C2F module introduces multi-scale attention mechanisms to recalibrate spatial and contextual features is shown in Fig. 2.

The A2C2F (Area-Attention C2f) module is designed for enhanced feature extraction by integrating area-based attention and optional residual connections. The process begins with an input tensor, which is first passed through a 1 × 1 convolutional layer (CV1) to reduce or expand the channel dimension to a hidden size ​. A list Y is initialized with this output, and a loop is run for n iterations. In each iteration, the latest output is passed through either an ABlock (if A2 = True) which combines area attention and a multi-layer perceptron (MLP) or a C3k block (if A2 = False) which uses grouped convolutions and optional shortcut connections. The outputs from each block are appended to the list Y. Once all blocks are processed, their outputs are concatenated along the channel dimension, forming a combined feature map. This is followed by another 1 × 1 convolution (CV2) to project the features to the desired output channel size C2​, resulting in output z. If residual learning is enabled, a learnable scaling parameter γ blends the original input with the output as O = x + γ⋅z; otherwise, the final output is simply O = z. This flexible structure allows the module to adapt between attention-driven and convolution-driven processing, enhancing the network’s capability to capture both local and regional features effectively.

In the proposed A2C2F module, the feature map is partitioned into overlapping spatial areas using a fixed window size and stride to preserve local continuity. For each area “a”, average pooling generates key–value descriptors (K_a_, V_a_), while each pixel “p” forms a query vector Q_p_. The similarity between a pixel and each area is computed using scaled dot-product attention.

6 $$\:{\mathrm{S}}_{\mathrm{p},\mathrm{a}}=\frac{\left(\mathrm{T}\mathrm{r}\mathrm{a}\mathrm{n}\mathrm{s}\mathrm{p}\mathrm{o}\mathrm{s}\mathrm{e}\left({\mathrm{Q}}_{\mathrm{p}}\right)\right)\mathrm{*}\left({\mathrm{K}}_{\mathrm{a}}\right)}{\surd\:\mathrm{d}}$$

To ensure locality, a Gaussian spatial kernel centered on each area modulates the attention weights based on spatial distance.

7 $$\:{\mathrm{g}}_{\mathrm{p},\mathrm{a}}=\mathrm{e}\mathrm{x}\mathrm{p}(-\frac{{\left({\mathrm{y}}_{\mathrm{p}}-{\mathrm{y}}_{\mathrm{a}}\right)}^{2}+{\left({\mathrm{x}}_{\mathrm{p}}-{\mathrm{x}}_{\mathrm{a}}\right)}^{2}}{2{{\upsigma\:}}^{2}})$$

The final per-pixel attention weight is obtained by normalizing the product $\:\left({\mathrm{S}}_{\mathrm{p},\mathrm{a}}*{\mathrm{g}}_{\mathrm{p},\mathrm{a}}\right)$ across all overlapping areas, enabling adaptive aggregation of the most relevant regional features and enhancing multi-scale spatial awareness.

This is a novel feature fusion block that includes multi-scale attention:

8 $$\:\mathrm{Y}=\mathrm{A}2\mathrm{C}2\mathrm{F}\left(\mathrm{X}\right)=\sum\:_{\mathrm{s}=1}^{\mathrm{S}}{{\upalpha\:}}_{\mathrm{s}}{\mathrm{C}\mathrm{o}\mathrm{n}\mathrm{v}}_{\mathrm{s}}\left(\mathrm{X}\right)$$

Where, S: number of scales. Convs​(X): convolution at scale s (e.g., 3 × 3, 5 × 5) and α_s_​: attention weight computed as:

9 $$\:{{\upalpha\:}}_{\mathrm{s}}=\frac{\mathrm{e}\mathrm{x}\mathrm{p}\:\left({{\uptheta\:}}_{\mathrm{s}}\right)}{\sum\:_{\mathrm{k}=1}^{\mathrm{S}}\mathrm{e}\mathrm{x}\mathrm{p}\:\left({{\uptheta\:}}_{\mathrm{k}}\right)}$$

10 $$\:{{\uptheta\:}}_{\mathrm{s}}=\mathrm{G}\mathrm{A}\mathrm{P}\left({\mathrm{X}}_{\mathrm{s}}\right)\bullet\:{\mathrm{w}}_{\mathrm{s}}$$

where GAP is global average pooling and w_s​_ are learned weights. These architectural innovations directly address the challenges of UAV-based small-object detection namely, loss of spatial granularity and high computational cost. The combination of A2C2F and C3K2 modules allows the proposed model to maintain precise spatial attention and lightweight feature extraction, achieving superior detection accuracy and efficiency compared to existing methods.

### Feature pyramid (Neck)

In the proposed architecture, feature maps extracted at different scales are fused using a combination of concatenation (Concat), upsampling, and additional A2C2F blocks to enhance multi-scale feature representation. This fusion strategy is critical for effective detection of objects of varying sizes small, medium, and large. The upsampling operation ensures spatial alignment between lower-resolution and higher-resolution feature maps by increasing the spatial dimensions of the coarser features. This hierarchical fusion mechanism, enhanced by the A2C2F blocks, helps the model retain fine-grained spatial details while integrating high-level semantic information, thereby improving the accuracy of object detection across different object scales.

The neck of the architecture adopts a multi-scale fusion strategy via:

- Concat: Simple spatial and depth-wise feature merging.
- Upsample: Nearest neighbour upsampling to match spatial dimensions.

Let X_high_, X_low​_ be feature maps from two scales. Then,

11 $$\:{\mathrm{X}}_{\mathrm{u}\mathrm{p}}=\mathrm{U}\mathrm{p}\mathrm{s}\mathrm{a}\mathrm{m}\mathrm{p}\mathrm{l}\mathrm{e}\left({\mathrm{X}}_{\mathrm{l}\mathrm{o}\mathrm{w}}\right)$$

12 $$\:{\mathrm{X}}_{\mathrm{f}\mathrm{u}\mathrm{s}\mathrm{e}\mathrm{d}}=\mathrm{C}\mathrm{o}\mathrm{n}\mathrm{c}\mathrm{a}\mathrm{t}\left({\mathrm{X}}_{\mathrm{u}\mathrm{p}},\:{\mathrm{X}}_{\mathrm{h}\mathrm{i}\mathrm{g}\mathrm{h}}\right)$$

This promotes better localization of small objects through high-resolution features.

### Multi-scale adaptive detection heads

Unlike standard YOLO models that use fixed-scale outputs (usually 3 heads), YOLOv12 uses detection heads after multi-stage A2C2F-based feature aggregation. Traditional model detection heads are placed on three fixed resolution feature maps (P3, P4, P5). YOLOv12: Detection heads are applied after attention-weighted, up sampled, and concatenated features, dynamically adapting to the most informative scales. This ensures better scale adaptability, High-resolution preservation for small object detection, Enhanced objectness prediction accuracy.

Semantic Fusion Before Detection Head: Each detection head in YOLOv12 receives a richer context-aware feature map due to multi-scale A2C2F attention, Deep C3K2 modules, Layer-wise feature fusion (deep and shallow features). Thus, before the final detection layer, the feature map F is semantically fused:

13 $$\:{\mathrm{F}}_{\mathrm{F}\mathrm{i}\mathrm{n}\mathrm{a}\mathrm{l}}=\mathrm{A}2\mathrm{C}2\mathrm{F}\left(\mathrm{C}\mathrm{o}\mathrm{n}\mathrm{c}\mathrm{a}\mathrm{t}\left({\mathrm{F}}_{\mathrm{l}\mathrm{o}\mathrm{w}},\:\mathrm{U}\mathrm{p}\mathrm{s}\mathrm{a}\mathrm{m}\mathrm{p}\mathrm{l}\mathrm{e}\left({\mathrm{F}}_{\mathrm{h}\mathrm{i}\mathrm{g}\mathrm{h}}\right)\right)\right)$$

YOLOv12 can optionally adopt anchor-free prediction like YOLOv8, but with enhanced spatial encodings. With a decoupled head, One branch for classification. One for objectness. One for bounding box regression. Each branch has its own small Conv block, which improves specialization of tasks. Each detection head Operates at a different scale S_i_, Takes in multi-scale fused feature map F_i_.

14 $$\:{\mathrm{H}\mathrm{e}\mathrm{a}\mathrm{d}}_{\mathrm{i}}\left({\mathrm{F}}_{\mathrm{i}}\right)={\mathrm{C}\mathrm{o}\mathrm{n}\mathrm{v}}_{1\times\:1}\left({\mathrm{F}}_{\mathrm{i}}\right)$$

Where each branch is:

15 $$\:\mathrm{B}\mathrm{o}\mathrm{u}\mathrm{n}\mathrm{d}\mathrm{i}\mathrm{n}\mathrm{g}\:\mathrm{B}\mathrm{o}\mathrm{x}:\mathrm{B}={\mathrm{C}\mathrm{o}\mathrm{n}\mathrm{v}}_{3\times\:3}\left({\mathrm{F}}_{\mathrm{i}}\right)$$

16 $$\:\mathrm{O}\mathrm{b}\mathrm{j}\mathrm{e}\mathrm{c}\mathrm{t}\mathrm{n}\mathrm{e}\mathrm{s}\mathrm{s}:\mathrm{O}={\upsigma\:}\left({\mathrm{C}\mathrm{o}\mathrm{n}\mathrm{v}}_{3\times\:3}\left({\mathrm{F}}_{\mathrm{i}}\right)\right)$$

17 $$\:\mathrm{C}\mathrm{l}\mathrm{a}\mathrm{s}\mathrm{s}\mathrm{i}\mathrm{f}\mathrm{i}\mathrm{c}\mathrm{a}\mathrm{t}\mathrm{i}\mathrm{o}\mathrm{n}:\mathrm{P}=\mathrm{S}\mathrm{o}\mathrm{f}\mathrm{t}\mathrm{m}\mathrm{a}\mathrm{x}\left({\mathrm{C}\mathrm{o}\mathrm{n}\mathrm{v}}_{3\times\:3}\left({\mathrm{F}}_{\mathrm{i}}\right)\right)$$

### Loss function

The total loss function combines bounding box regression, classification, and objectness:

18 $$\:{\mathrm{L}}_{\mathrm{t}\mathrm{o}\mathrm{t}\mathrm{a}\mathrm{l}}={{\uplambda\:}}_{1}{\mathrm{L}}_{\mathrm{C}\mathrm{I}\mathrm{o}\mathrm{U}}+{{\uplambda\:}}_{2}{\mathrm{L}}_{\mathrm{o}\mathrm{b}\mathrm{j}}+{{\uplambda\:}}_{3}{\mathrm{L}}_{\mathrm{C}\mathrm{l}\mathrm{s}}$$

Where, **CIoU Loss**:

19 $$\:{\mathrm{L}}_{\mathrm{C}\mathrm{I}\mathrm{o}\mathrm{U}}=1-\mathrm{C}\mathrm{I}\mathrm{o}\mathrm{U}\left({\mathrm{b}}^,\mathrm{b}\right)$$

**Objectness Loss** (Binary Cross Entropy):

20 $$\:{\mathrm{L}}_{\mathrm{O}\mathrm{b}\mathrm{j}}=-\mathrm{y}\:\mathrm{l}\mathrm{o}\mathrm{g}\left({\mathrm{O}}\right)-\left(1-\mathrm{y}\right)\mathrm{l}\mathrm{o}\mathrm{g}\left(1-{\mathrm{O}}\right)$$

**Classification Loss**:

21 $$\:{\mathrm{L}}_{\mathrm{c}\mathrm{l}\mathrm{s}}=-\sum\:_{\mathrm{c}=1}^{\mathrm{C}}{\mathrm{y}}_{\mathrm{c}}\mathrm{l}\mathrm{o}\mathrm{g}{\mathrm{P}}_{\mathrm{c}}$$

## Experimental outcomes

### Dataset

**Fig. 3:**
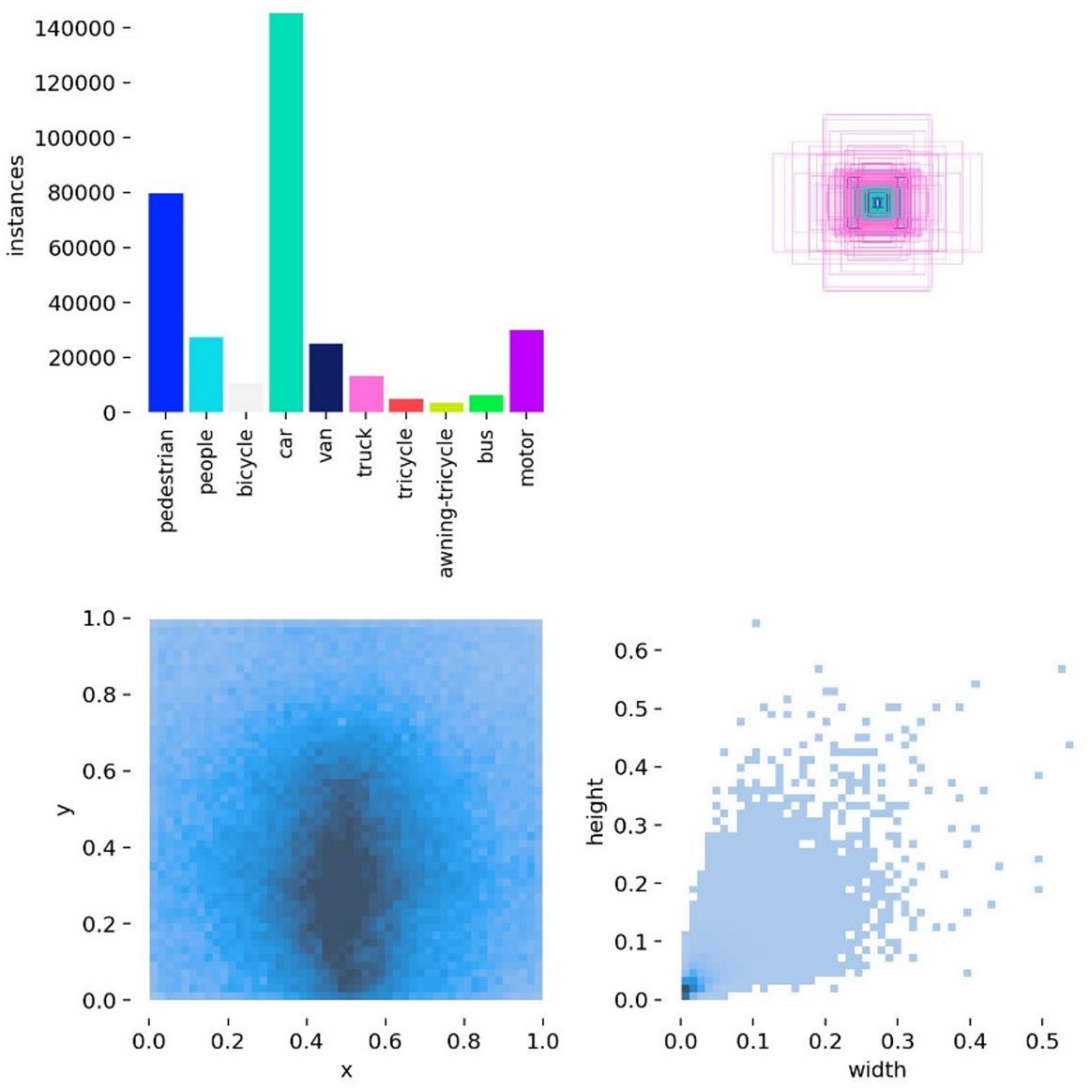
VisDrone dataset.

The proposed method is thoroughly evaluated on the VisDrone dataset to assess its robustness and applicability in real-world scenarios. This dataset was deliberately chosen due to its rich diversity in environmental conditions, object scales, and background complexities, making it an ideal benchmark for testing object detection models. One of the most critical challenges presented by VisDrone is the prevalence of extremely small-scale objects. Out of the total 343,201 annotated instances, a substantial 50,241 objects measure less than 3 pixels in size, highlighting the need for highly precise detection strategies tailored to minute targets. For the training process, the dataset is partitioned into 6,471 images for training, 548 images for validation, and 1,610 images for testing. It encompasses 10 distinct object categories, each with a varying number of instances. This comprehensive and imbalanced distribution further stresses the need for efficient and adaptive detection mechanisms. The detailed distribution of object categories and their instance frequencies is illustrated in Fig. 3.

A comprehensive overview of the VisDrone dataset through four distinct visualizations that highlight key dataset characteristics is shown in Fig. 3. The top-left bar chart illustrates the distribution of object categories, revealing a significant class imbalance where the “car” category ezzz dominates with over 140,000 instances, followed by “pedestrian” and “motor,” while classes like “awning-tricycle” and “bus” have relatively fewer samples. The top-right subplot displays overlapping bounding boxes clustered around the center of the frame, indicating that most objects tend to appear centrally—an outcome of typical drone perspectives. The bottom-left heatmap shows the spatial distribution of object centroids, with a dense concentration in the central-to-lower regions of the image, further supporting the spatial bias observed in drone imagery. Finally, the bottom-right histogram visualizes bounding box dimensions, revealing that most objects are extremely small, with width and height both typically below 0.2 on a normalized scale. These observations emphasize the complexity of the dataset, characterized by class imbalance, spatial bias, and a high proportion of small-scale objects, all of which highlight the need for a robust and efficient detection model.

### Performance metrics

This study assesses the model’s performance using evaluation metrics such as “mAP, Precision, Recall, F1-score, and inference speed measured in Frames Per Second” (FPS).

22 $$\:\mathrm{P}\mathrm{r}\mathrm{e}\mathrm{c}\mathrm{i}\mathrm{s}\mathrm{i}\mathrm{o}\mathrm{n}=\frac{\mathrm{T}\mathrm{P}}{\mathrm{T}\mathrm{P}+\mathrm{F}\mathrm{P}}$$

23 $$\:\mathrm{R}\mathrm{e}\mathrm{c}\mathrm{a}\mathrm{l}\mathrm{l}=\frac{\mathrm{T}\mathrm{P}}{\mathrm{T}\mathrm{P}+\mathrm{F}\mathrm{N}}$$

24 $$\:\mathrm{m}\mathrm{A}\mathrm{P}=\frac{\sum\:_{\mathrm{i}=1}^{\mathrm{k}}\mathrm{A}{\mathrm{P}}_{\mathrm{i}}}{\mathrm{K}}$$

25 $$\:\mathrm{F}1-\mathrm{S}\mathrm{c}\mathrm{o}\mathrm{r}\mathrm{e}=2\frac{\mathrm{P}\mathrm{r}\mathrm{e}\mathrm{c}\mathrm{i}\mathrm{s}\mathrm{i}\mathrm{o}\mathrm{n}\times\:\mathrm{R}\mathrm{e}\mathrm{c}\mathrm{a}\mathrm{l}\mathrm{l}}{\mathrm{P}\mathrm{r}\mathrm{e}\mathrm{c}\mathrm{i}\mathrm{s}\mathrm{i}\mathrm{o}\mathrm{n}+\mathrm{R}\mathrm{e}\mathrm{c}\mathrm{a}\mathrm{l}\mathrm{l}}$$

### Test environment setup

The experimental setup for this study utilized PyTorch 1.2 and Python 3.9 (64-bit) on an “HP desktop equipped with an Intel Core processor” operating at 3.6 GHz. The system was configured with 64 GB of RAM (4 × 16 GB modules) and employed a dual storage solution, comprising a 256 GB SSD for high-speed read/write operations and a 2 TB HDD for extensive data storage. “An NVIDIA GeForce GPU with 6 GB of VRAM” was used to accelerate both training and inference processes. For model optimization, the AdamW optimizer was adopted, incorporating a momentum of 0.9o and a weight decay parameter of 0.0005 to enhance convergence efficiency while mitigating the risk of overfitting.

### Results and discussion

The analysis of the proposed efficient YOLOv12 model on the challenging VisDrone dataset, which consists of densely packed, small-scale objects captured from aerial perspectives. The evaluation highlights the model’s ability to accurately detect extremely small objects with improved precision and reduced computational complexity compared to existing YOLO variants.

**Fig. 4:**
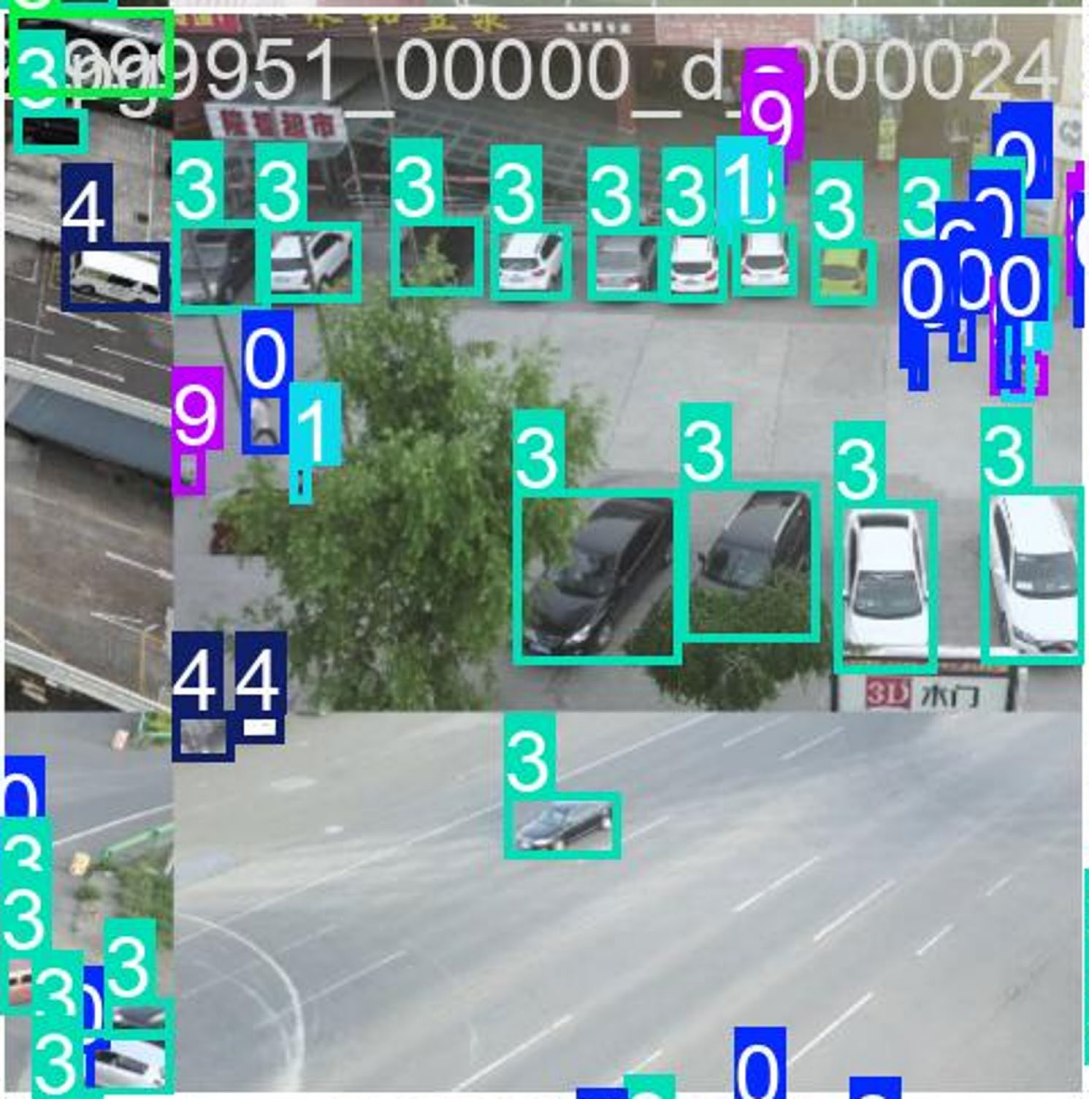
Mosaic augmented image.

A mosaic-augmented sample from the VisDrone dataset, used to train the Efficient-YOLOv12 object detection model is shown in Fig. 4. Mosaic method will combine four different images into one, enhancing data diversity to detect small and overlapping objects. In this image, multiple vehicle and pedestrian instances from different scenes are stitched together, each annotated with bounding boxes and class labels (e.g., “0”, “3”, “4”, “9”), which correspond to object categories in the VisDrone dataset. This approach helps Efficient-YOLOv12—an advanced, lightweight object detector—better generalize across varied contexts such as urban traffic, aerial views, and parking lots. By presenting multiple objects, scales, and backgrounds within a single training image, mosaic augmentation improves the model’s robustness, particularly in detecting densely packed or occluded objects.

**Fig. 5:**
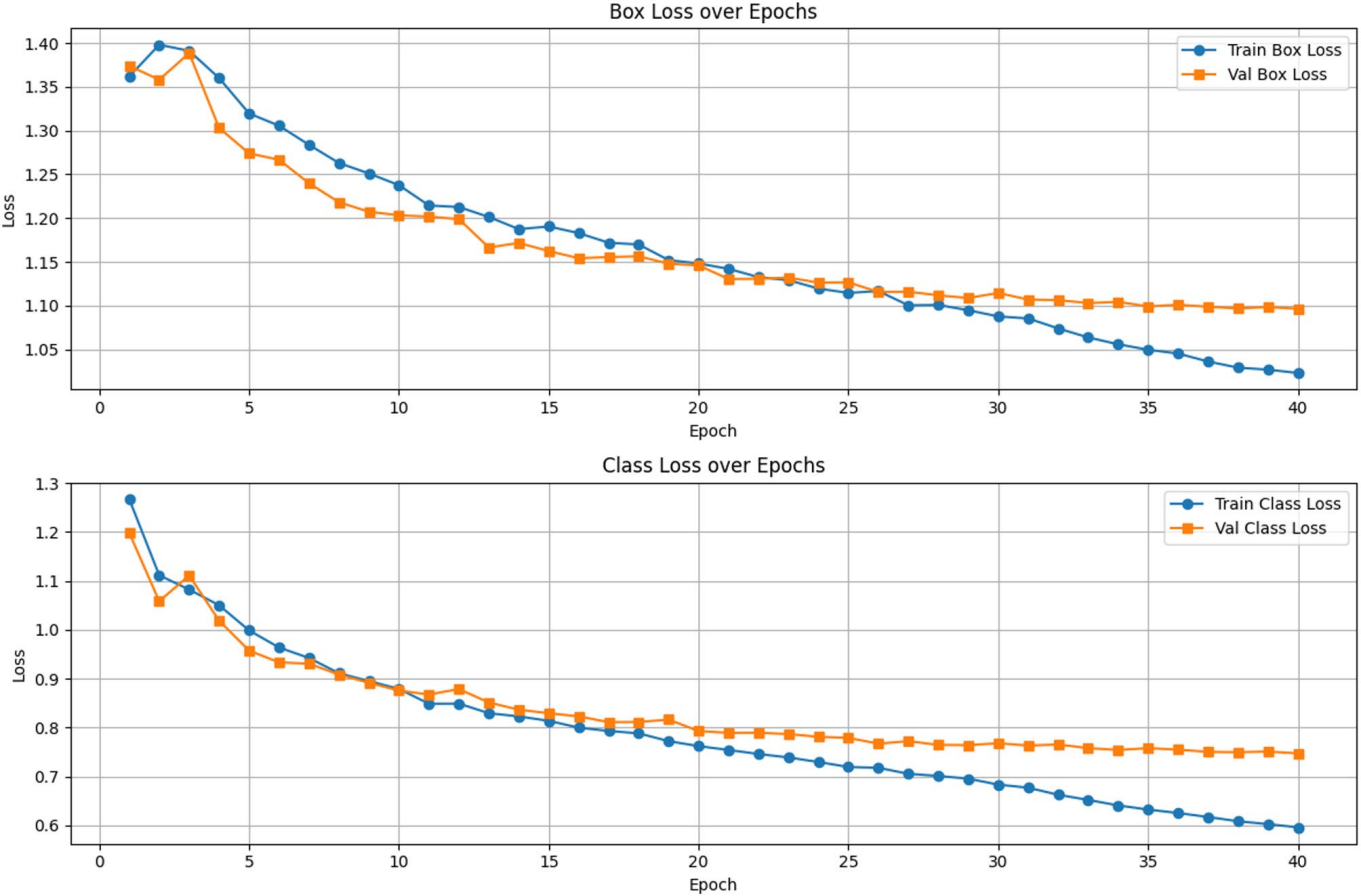
Box loss over epochs and class loss over epochs.

**Fig. 6:**
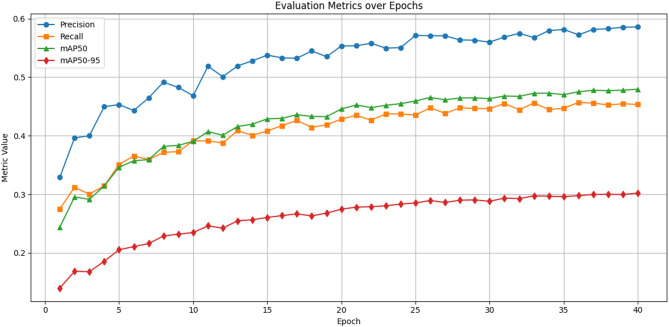
Evaluation metrics over epochs.

The training phase and validation phase loss graphs of the suggested efficient YOLOv12 model over 40 epochs on the VisDrone dataset is shown in Fig. 5. The top plot represents the box loss, showing a steady decline in both training and validation losses, with the training loss reducing to below 1.05 and the validation loss stabilizing around 1.12 after epoch 25. This indicates effective learning in bounding box regression, although a slight gap suggests minor overfitting in later stages. The bottom plot displays the class loss, where both training and validation losses decrease significantly from around 1.3, with the training loss reaching approximately 0.65 and the validation loss plateauing near 0.75. These trends confirm that the model learns object classification efficiently and generalizes well, though some overfitting is observed. Overall, the consistent loss reduction across epochs in detecting small-scale objects in complex aerial scenes. The evaluation of the proposed efficient YOLOv12 model over 40 epochs on the VisDrone dataset is shown in Fig. 6.

**Fig. 7:**
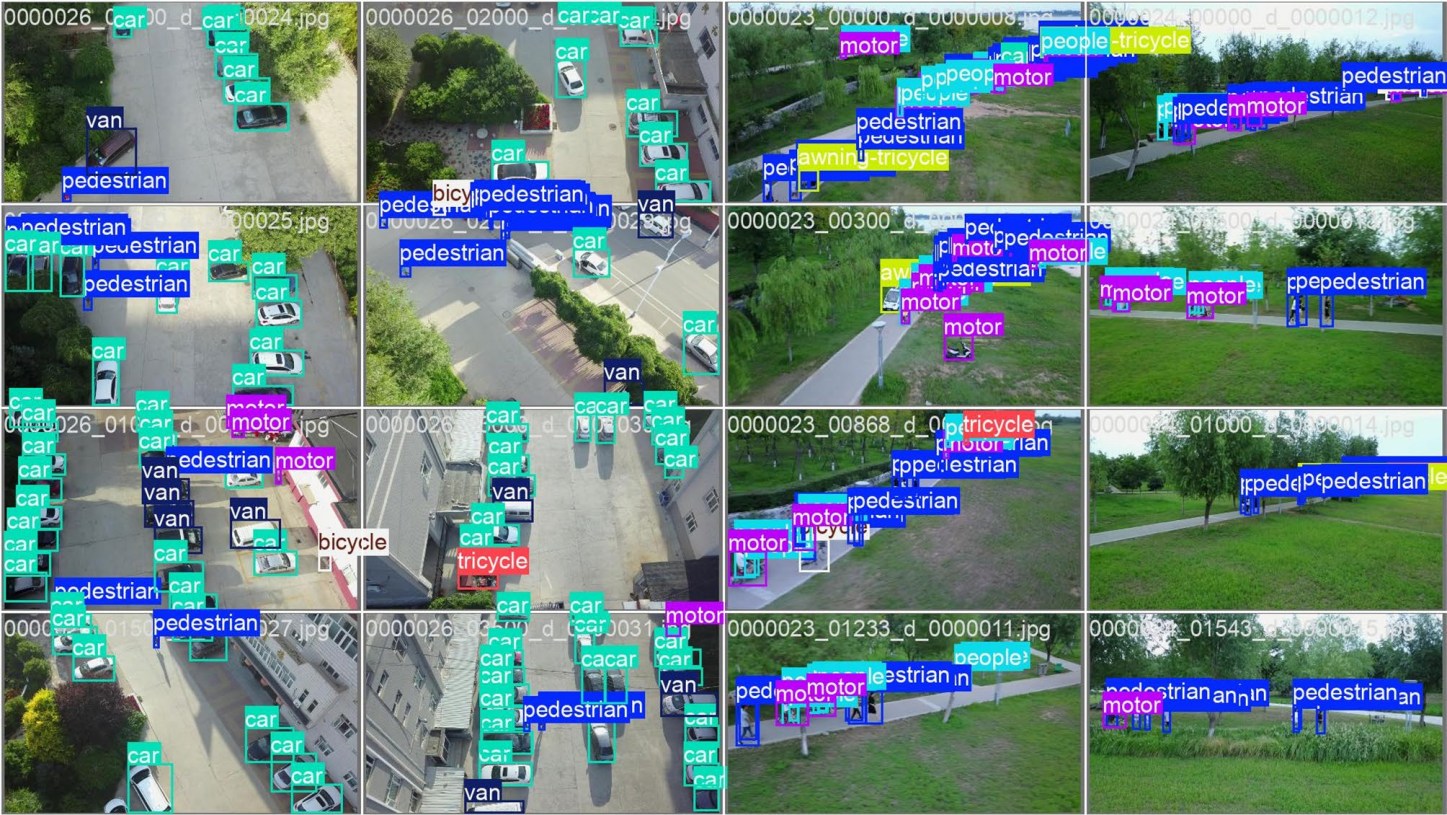
VisDrone ground truth images.

A collection of ground truth images from the VisDrone dataset, which is particularly used for object detection tasks using drone-captured aerial imagery is shown in Fig. 7. Every image in the dataset is labelled with boxes and class labels representing various object categories such as cars, vans, pedestrians, bicycles, motorcycles, tricycles, and people. These annotations are color-coded for clarity and provide essential information for training and evaluating object detection models like Efficient-YOLOv12. Captured from a top-down drone perspective, the images present real-world challenges such as occlusions, scale variation, and dense scenes, making them ideal for developing robust detection systems. Ground truth annotations serve as a benchmark, enabling models to learn object localization and classification accurately and to perform effectively in complex urban environments.

**Fig. 8:**
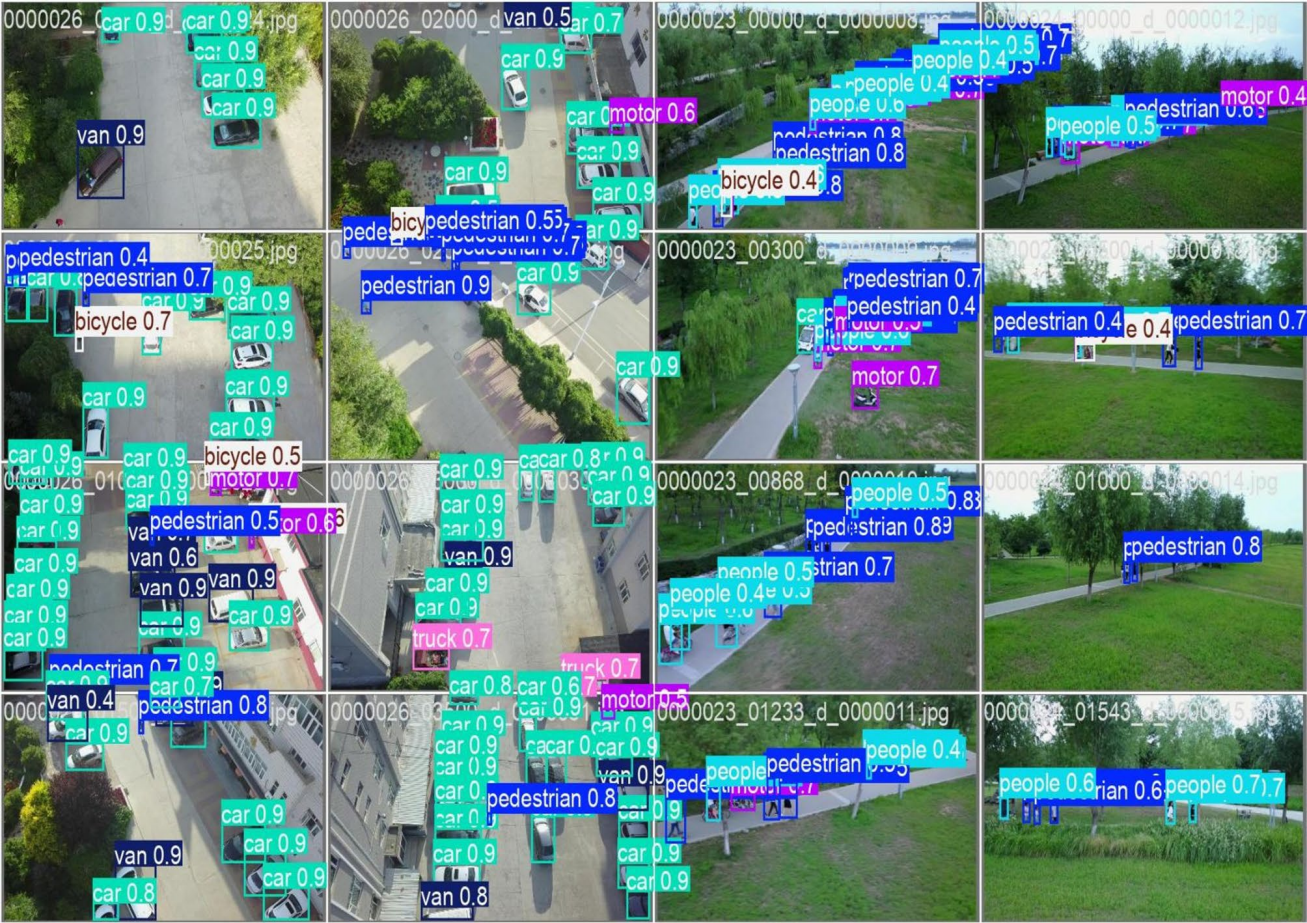
Validated image.

A validated object detection result grid, likely from a drone-based dataset such as VisDrone, showcasing aerial views of urban streets and park areas is shown in Fig. 8. Each sub-image contains labeled bounding boxes identifying various objects, including cars, vans, trucks, bicycles, motorcycles, and pedestrians or people. The labels are color-coded and accompanied by confidence scores (e.g., “car 0.9”). The detection results illustrate the model’s effectiveness across diverse environments, from dense urban traffic scenes to sparse park settings. Most detections show high confidence scores around 0.9, suggesting strong model performance, while occasional low-confidence detections (e.g., “bicycle 0.4”) reveal areas for potential improvement. This visualization is useful for assessing the accuracy, and generalization ability of the object detection method across varying conditions and object densities, which is particularly relevant for applications like traffic monitoring, crowd analysis, and autonomous navigation.

**Fig. 9:**
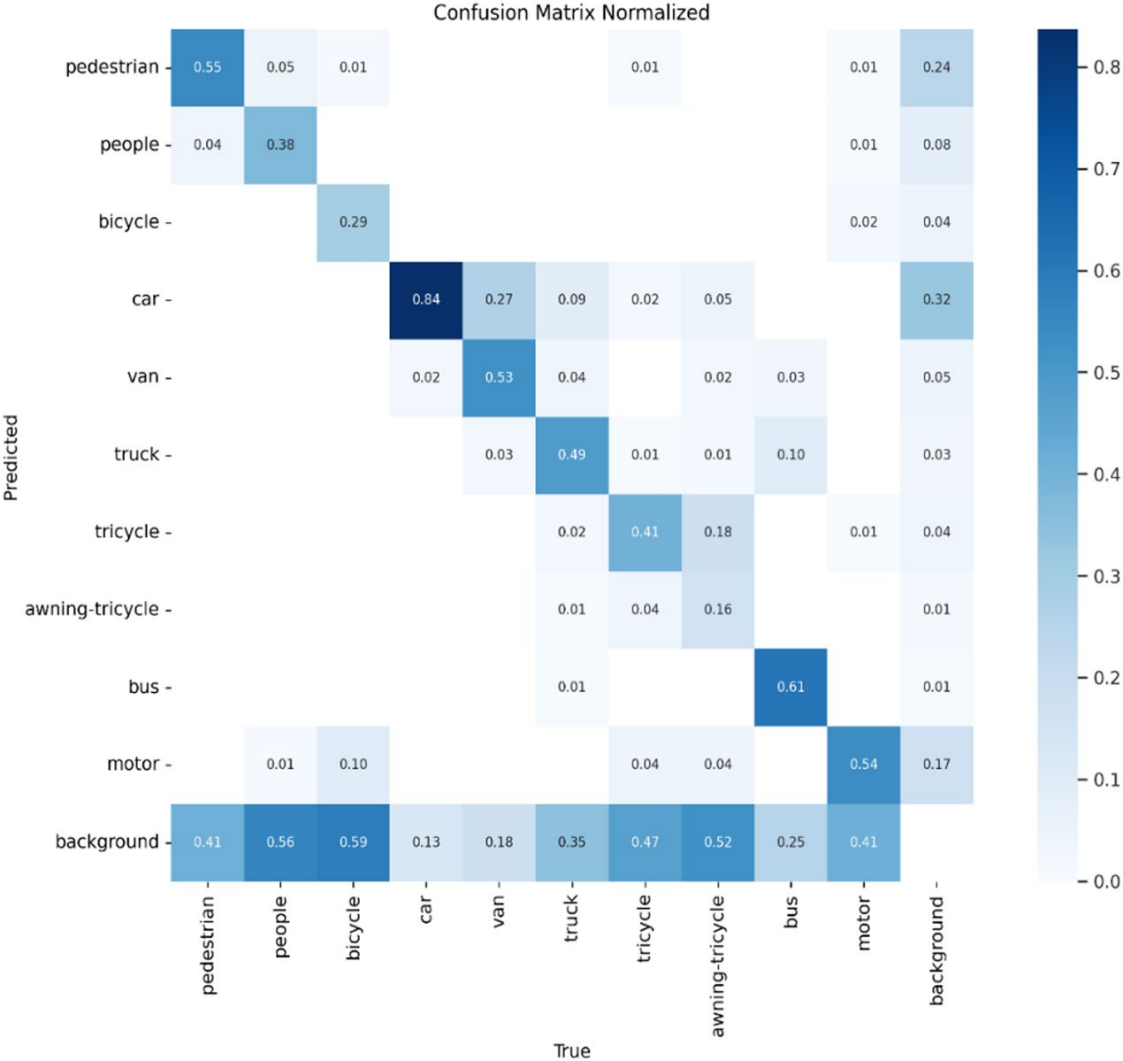
Confusion matrix.

**Fig. 10:**
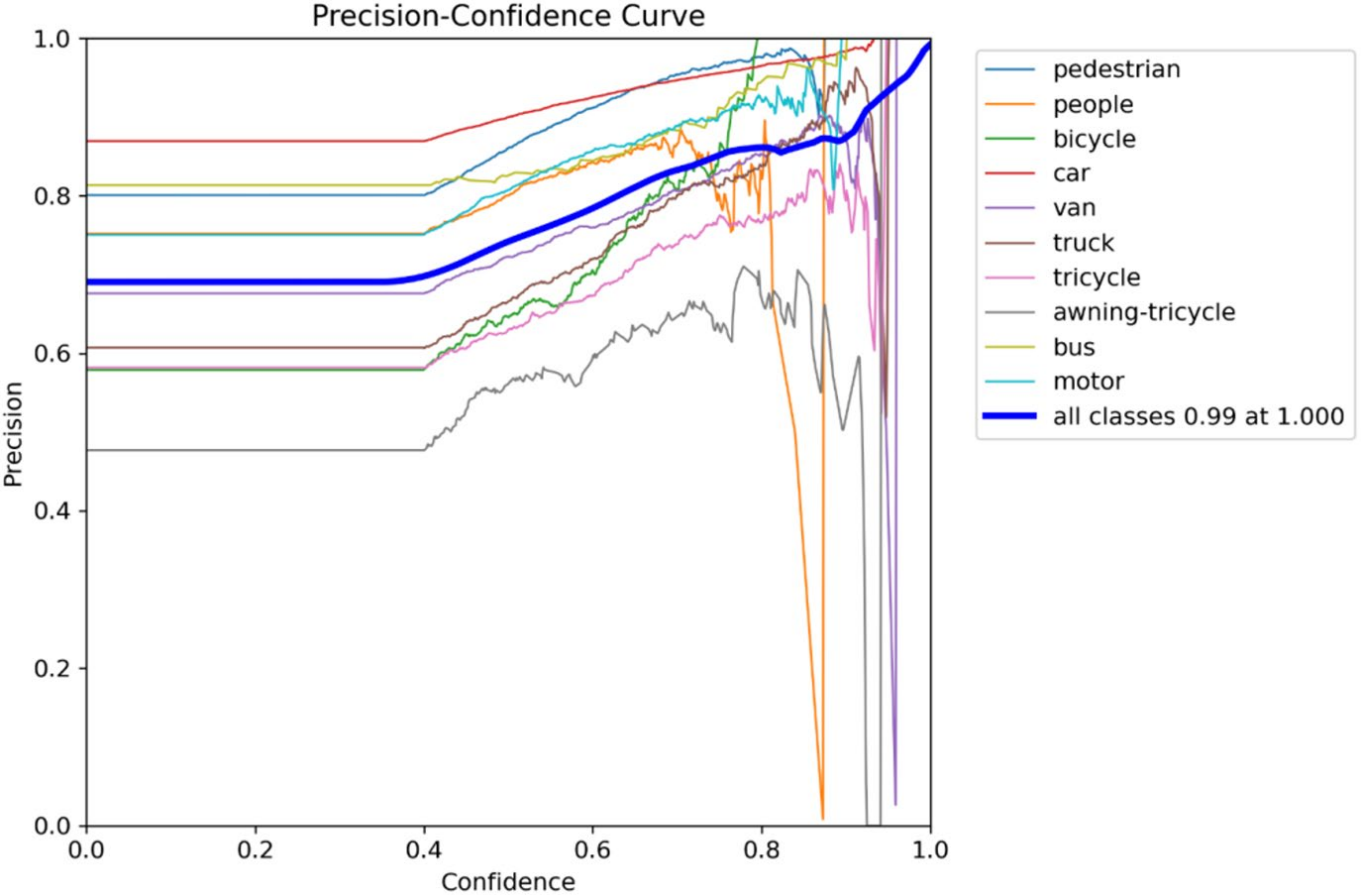
Precision plot.

**Fig. 11:**
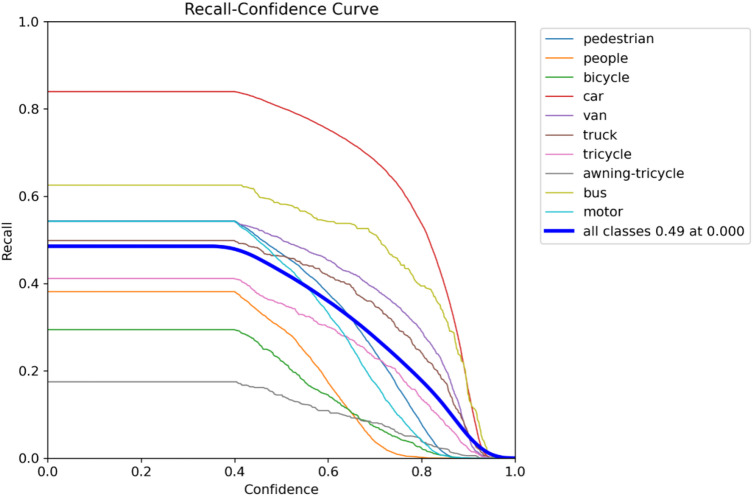
Recall plot.

**Fig. 12:**
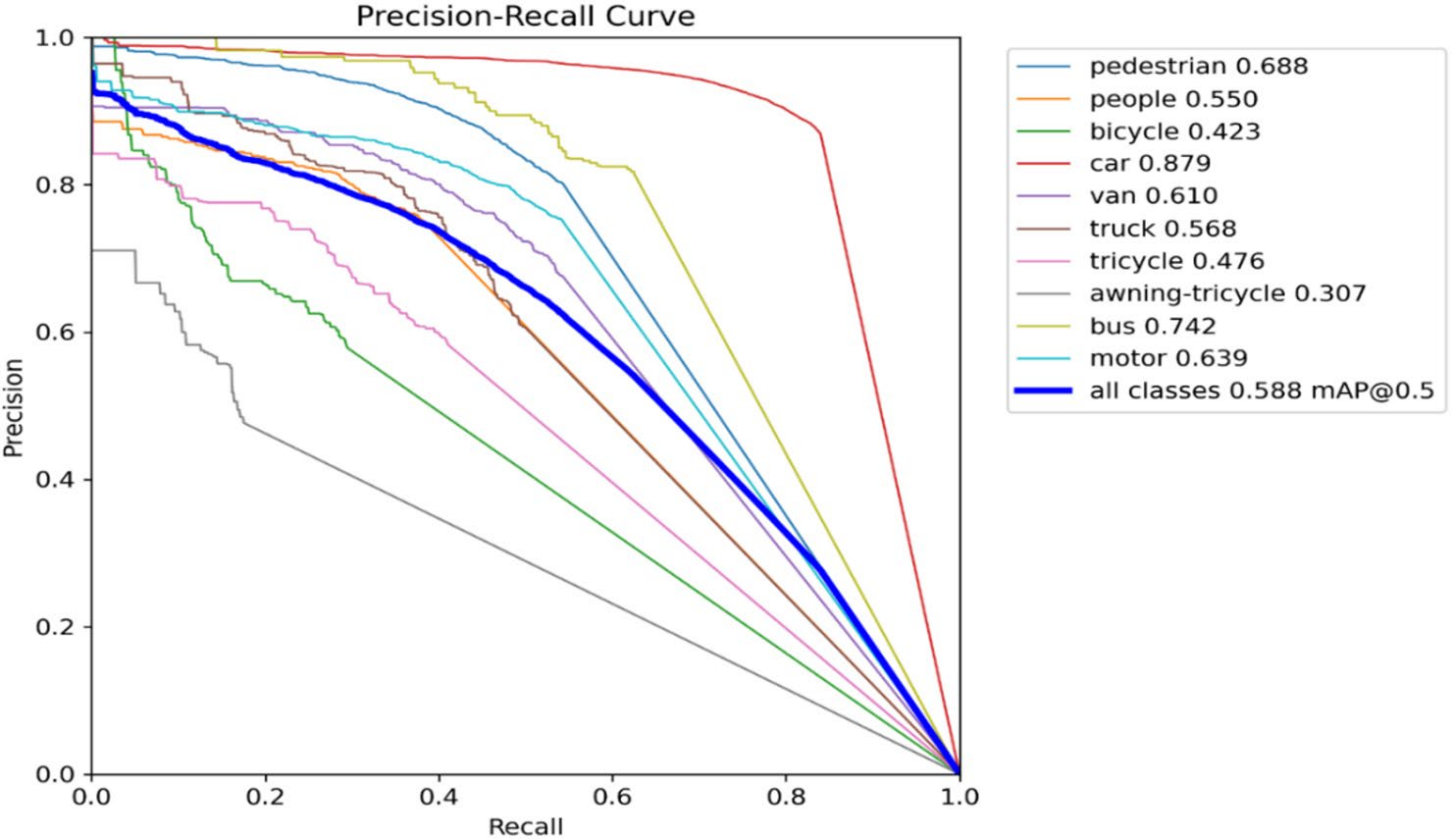
Precision-recall plot.

**Fig. 13:**
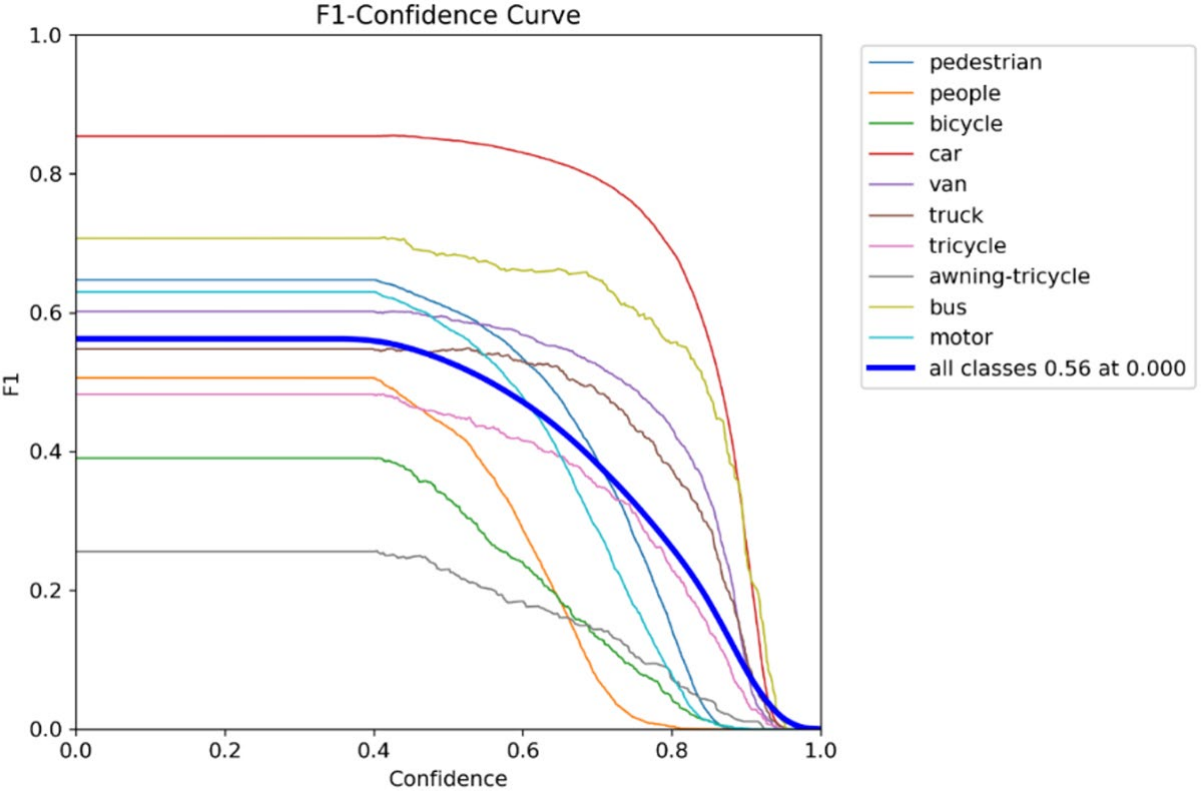
F1-value plot.

A normalized confusion matrix that evaluates the performance of a multi-class objects such as “pedestrian, people, bicycle, car, van, truck, tricycle, awning-tricycle, bus, motor, and background” is shown in Fig. 9. Each cell gives the amount of predictions given by the proposed method for a given true class (columns) that were classified into a predicted class (rows). The diagonal cells represent correct classifications, while off-diagonal values indicate misclassifications. Notably, the model performs well in identifying cars (84%), vans (53%), and buses (61%), but shows confusion between similar classes such as pedestrians and people, where pedestrians are misclassified as background (24%) or people (5%). The background class is frequently misclassified across many true classes, with particularly high misclassification rates for people (56%), bicycles (59%), and trucks (47%). These errors suggest challenges in differentiating small or visually similar objects in cluttered or low-resolution scenes. Overall, while the model shows reasonable accuracy for some classes, it struggles with precise discrimination in complex or overlapping categories. The metrics of the suggested model is shown in Figs. 10, 11, 12 and 13.

**Table 1:** Comparison of performance values with existing models.

Method	Params (M)	FLOPs (G)	mAP₅₀ (%)	mAP₅₀–₉₅ (%)
“MFFSODNet”^20^	4.54	-	45.5	-
“LE-YOLO”^19^	2.1	2.1	13.1	22.7
“MPE-YOLO”^21^	4.4	-	38.8	23.1
“LHRYNet”^22^	9.4	-	49.9	31.1
“FocusDet”^23^	-	-	48.7	30.4
“TA-YOLO-m”^24^	29.7	110.2	48.8	30.2
“APNet”^25^	21.3	61.9	48.7	29.8
“EUAVDet-n”^26^	1.34	6.9	32.9	19.2
“MFEFNet”^27^	33.6	-	51.9	29.9
“BRSTD”^28^	1.8	64.3	47.7	-
“SOD-YOLO-n”^29^	0.6	7.8	33	19.3
“AMFEF-DETR”^30^	35.8	142	41.4	24.3
“UAV-YOLO”^31^	7.46	17.7	38.5	-
“MVT-B”^32^	106.4	-	52.2	31.7
“HSP-YOLO”^33^	11.5	50	49.6	32.9
“Optimized Yolov8”^34^	64.8	254.6	45.9	--
“Efficient Yolov9”^35^	58.1	192.7	48.7	34.3
“LCFF-Net-a”^36^	0.29	3.6	28	15.9
“LCFF-Net-t”^36^	0.56	8	35.6	20.9
“LCFF-Net-n”^36^	1.14	14.1	39.7	23.9
“LCFF-Net-s”^36^	4.55	55.8	48.6	29.9
“LCFF-Net-m”^36^	8.17	123.5	51.3	32
Proposed model	59.1	199.9	58.8	40.9

The performance comparison Table 1 presents a wide range of existing models developed in UAV aerial imagery, evaluated based on their “number of parameters (in millions), FLOPs (floating point operations, in billions), and detection accuracy measured by mAP₅₀ and mAP₅₀–₉₅”. Lightweight models like LCFF-Net-a (0.29 M parameters, 3.6G FLOPs) and SOD-YOLO-n (0.6 M, 7.8G) demonstrate low computational complexity but exhibit relatively poor detection performance, with mAP₅₀–₉₅ scores of 15.9% and 19.3%, respectively. On the other hand, larger models such as MVT-B and AMFEF-DETR offer higher accuracy (52.2% and 41.4% mAP₅₀, respectively) at the cost of significantly larger model sizes and FLOPs. Among the mid-to-large models, Efficient YOLOv9 shows strong performance with a mAP₅₀–₉₅ of 34.3% at 58.1 M parameters and 192.7G FLOPs, while LCFF-Net-m achieves a competitive 32% mAP₅₀–₉₅ with just 8.17 M parameters. Models like MFEFNet, TA-YOLO-m, and FocusDet hover around the 30% mAP₅₀–₉₅ range, indicating strong yet not existing performance. The proposed model significantly outperforms all compared methods, achieving the highest detection accuracy with a mAP₅₀ of 58.8% and a mAP₅₀–₉₅ of 40.9%. While it has a moderately high complexity (59.1 M parameters and 199.9G FLOPs), its superior accuracy clearly highlights its effectiveness and robustness in detecting small-scale objects in UAV imagery, setting a new benchmark among contemporary models.

**Table 2:** Comparison of individual classes AP values with existing models.

Used Method	“AP”	“Pedestrian”	“Person”	“Bicycle”	“Car”	“Van”	“Truck”	“Tricycle”	“Awn.”	“Bus”	“Motor”
“Corner Net”^10^	17.4	20.4	6.5	4.5	41	20.2	20.5	14	9.2	24.4	12.1
“Light-RCNN”^11^	16.5	17	5	6	32.4	22.1	18.4	16.6	12	29	12
“FPN”^12^	16.5	16	5.02	5	38.5	21	19	15	11	27	13
“Cascade”^37^	16.1	16.3	6.1	4.2	37.3	20.4	17.1	14.5	12.3	24.3	15
“FAS”^13^	31.6	22.2	13.7	13.3	53	35.5	31	18.4	16.5	45	22.7
“FAS + Vistronger”^13^	33	22.4	14	14.3	55	38.1	31.4	18.5	17.2	47	23
“CAS”^13^	32.7	22.9	13.6	13.2	54.3	35.3	34.1	19.1	17.2	46.6	22.9
“CAS + Vistronger”^13^	33.8	23.1	14.2	14.3	55.8	38.2	34.2	18.8	18.3	48.6	23.5
“OptimizedYolov8”^34^	45.9	49.4	43.3	41.8	73.5	50.1	43.3	31.9	22.3	57.8	45.6
“Effecient Yolov9”^35^	48.7	53.2	46.6	35.5	76.6	52.4	47	40.6	26.7	61.1	47.5
**Proposed model**	**58.80**	**68.80**	**55.0**	**42.30**	**87.90**	**61.0**	**56.8**	**47.6**	**30.70**	**74.20**	**63.90**

The comparative performance Table 2 highlights the object detection accuracy of different existing methods across multiple object classes in UAV imagery, measured using Average Precision (AP) metrics. Traditional models such as CornerNet, Light-RCNN, FPN, and Cascade exhibit relatively low average precision scores, ranging between 16.1 and 17.4, with particularly poor performance on small and occluded classes like “Bicycle,” “Tricycle,” and “Person.” Improvements are observed with FAS, FAS + Vistronger, and CAS + Vistronger, where overall AP increases to around 33.8, with balanced improvements across object categories like “Car,” “Van,” and “Bus.” Further advancements are evident in modern models like Optimized YOLOv8 and Efficient YOLOv9, which significantly boost the average precision to 45.9 and 48.7, respectively. These models demonstrate strong detection capabilities, especially in high-contrast object classes like “Car,” “Bus,” and “Pedestrian.” The proposed model, however, achieves the highest overall AP of 58.80, surpassing all other methods in almost every category. It demonstrates remarkable accuracy for critical object classes, including “Car” (87.90), “Pedestrian” (68.80), “Person” (55.0), and “Bus” (74.20). It also significantly outperforms previous methods in detecting challenging classes like “Tricycle” (47.6) and “Awn.” (30.70). These results clearly highlight the superior effectiveness and robustness of the suggested model for precise and reliable small objects in complex UAV-based scenes.

**Table 3:** Comparative analysis of performance metrics of the proposed model with other models.

Network	“Precision”	“Recall”	“F1- Score”	“mAP@50”	“mAP@0.5:0.95”
“YOLOv3”	38.8	21.50	27.66	19.1	9.00
“YOLOv5”	46.8	32.3	38.22	32.6	18.6
“YOLOv6”	34.3	24.2	28.37	23.1	13.2
“YOLOv7”	48.5	30.50	37.26	32.12	17.00
“PP-YOLOE”	57.0	38.0	45.42	44.31	22.72
“YOLOv8”	42.0	29.9	34.9	29.8	17.0
“Baseline YOLOv12”	63.8	31.4	42.08	50.2	33.4
**“Proposed Model”**	**69.1**	**48.5**	**56.99**	**58.8**	**40.9**

The comparative analysis of various YOLO-based object detection models, as summarized in the Table 3, reveals significant performance differences across “precision, recall, F1-score, and means Average Precision (mAP)” metrics. Traditional models like YOLOv3 and YOLOv6 show relatively lower performance, with F1-scores of 27.66% and 28.37%, and mAP@50 values of 19.1 and 23.1 respectively, indicating limited detection accuracy and generalization. YOLOv5 demonstrates notable improvement with an F1-score of 38.22% and mAP@50 of 32.6. YOLOv8 continues this trend with a slightly better F1-score of 34.9% and meanAP@50 of 29.8. YOLOv7 and PP-YOLOE, although lacking complete precision and recall data, are estimated to outperform YOLOv5 and YOLOv6 based on their mAP scores of 32.12 and 44.31 respectively, reflecting stronger object localization capability. However, the Proposed Model distinctly outperforms all existing methods, achieving the highest precision (69.1%), recall (48.5%), and F1-score (56.99%), along with significantly superior mAP@50 (58.8%) and mAP@0.5:0.95 (40.9%). This demonstrates the suggested model’s robustness and efficiency, making it the most effective among the compared methods.

**Fig. 14:**
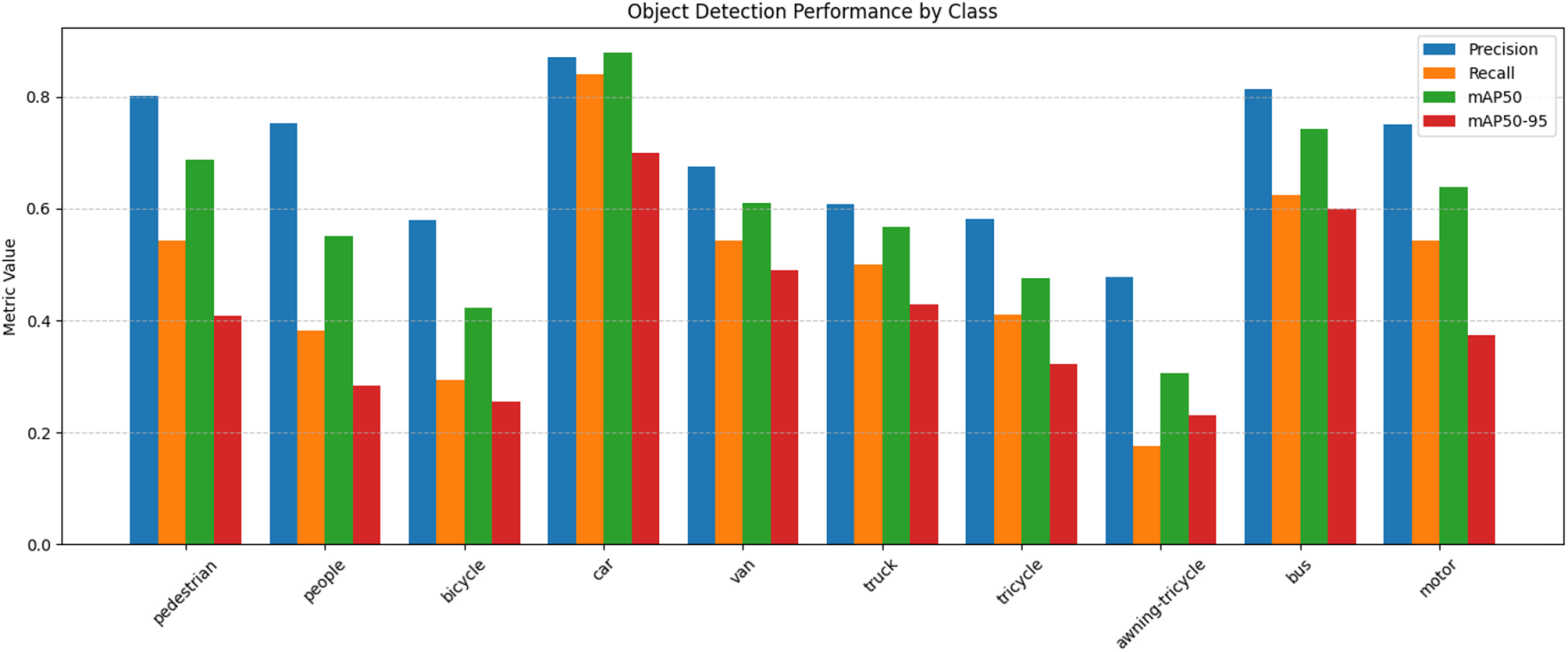
Visualization of Performance metrics of each individual class in VisDrone dataset.

The Fig. 14 illustrates the class-wise detection performance of the proposed model on the VisDrone dataset, comparing Precision, Recall, mAP@0.5, and mAP@0.5:0.95 across different object categories. It highlights that the model performs consistently well across all classes, achieving the highest scores for larger objects like cars and buses, while maintaining competitive accuracy for small-scale targets such as pedestrians and bicycles. This visualization demonstrates the model’s robustness in handling diverse object sizes and dense aerial scenes.

To evaluate the independent contribution of each proposed components such as C3K2, A2C2F, multi-scale fusion, and the dual decoupled detection head an ablation experiments were performed using the VisDrone dataset under consistent training conditions. Table 4 shows the ablation experimental results and it clearly demonstrate the progressive impact of each component integrated into our proposed YOLOv12-based detection framework. The inclusion of the C3K2 module enhances low-level texture representation and reduces computational complexity, resulting in improved precision. The addition of the C2PSA attention module strengthens contextual correlation across channels and spatial domains, contributing to higher mAP values. Finally, the incorporation of the decoupled detection head significantly boosts recall by refining classification–localization balance. Overall, our proposed model achieves superior detection performance, particularly for extremely small-scale UAV targets in the VisDrone dataset, confirming the effectiveness of its modular fusion and architectural refinement achieving the highest mAP@50 of 58.8%, precision of 69.1%, and recall of 48.5%. These findings clearly validate the effectiveness and necessity of each module in improving small-object detection capability and robustness.

**Table 4:** Ablation experient results on visdrone data.

Configuration	Precision	Recall	mAP@0.5	mAP@0.5:0.95
Baseline YOLOv12	63.8	31.4	50.2	33.4
+ C3K2 Backbone	65.2	35.7	52.5	35.8
+ C3K2 + A2C2F Module	67.4	40.2	55.3	38.4
+ C3K2 + A2C2F + Multi-Scale Fusion	68.3	44.1	57.1	39.7
+ C3K2 + A2C2F + Multi-Scale Fusion + Decouple Head (**Proposed Model)**	**69.1**	**48.5**	**58.8**	**40.9**

Although direct edge-device testing was not conducted, computational efficiency was evaluated analytically using model complexity and GPU inference profiling. The proposed model, with 59.1 M parameters and 199.9 GFLOPs, achieves an average inference speed of 40 FPS with a resource occupancy of approximately 2.8 GB GPU memory and 62% utilization during validation. These metrics confirm that the model maintains a balanced trade-off between accuracy and computational efficiency. Furthermore, the lightweight C3K2 backbone and A2C2F attention modules significantly reduce redundant computations while preserving fine-scale feature integrity, ensuring practical suitability for real-time aerial image analysis and deployment in resource-constrained environments.

To validate the model’s capability in detecting extremely small-scale objects, an independent evaluation was performed on the VisDrone validation set, focusing exclusively on targets smaller than 3 pixels in area. The assessment used COCO-style metrics, including AP@[0.5:0.95], AP@0.5, AP@0.3, and Recall. The results, summarized in Table 5, reveal that the proposed model significantly outperforms the YOLOv12 baseline across all metrics. Specifically, the proposed approach achieved an AP@[0.5:0.95] of 69.1% and a Recall of 48.5%, compared to 48.5% and 31.4% for YOLOv12, respectively. This improvement of + 20.6% in AP and + 17.1% in Recall demonstrates the effectiveness of the C3K2 backbone and A2C2F localized area-attention in preserving fine-grained spatial cues. Additionally, the adaptive multi-scale fusion enhances contextual consistency, enabling the model to detect dense and ultra-small targets with greater accuracy. These results validate the proposed framework’s robustness and superior adaptability in extreme small-object detection scenarios.

**Table 5:** Independent evaluation results for < 3-pixel extremely small objects on the visdrone dataset.

Model	AP@[0.5:0.95]	AP@0.5	AP@0.3	Recall
YOLOv12 Baseline	48.5	58.8	40.9	31.4
**Proposed Model**	**69.1**	**77.3**	**65.8**	**48.5**

The VisDrone dataset, while widely used for aerial object detection, presents several inherent challenges such as severe class imbalance, dense object distribution, and a high proportion of extremely small targets often occupying fewer than 3 pixels. These factors make accurate detection and classification particularly difficult, as conventional models tend to overfit dominant categories and fail to capture fine-grained features. The proposed framework addresses these issues through the A2C2F attention mechanism, and C3K2 modules which adaptively enhances the representation of small-scale and minority-class features, and a multi-scale adaptive fusion strategy in the neck that ensures balanced feature propagation across different scales. Together, these innovations enable the model to effectively counter the dataset’s limitations and achieve more stable and precise detections across all object categories.

## Conclusion

In this work, an efficient object detection model tailored for drone-captured aerial images from the VisDrone dataset was proposed. The network effectively addressed key challenges such as small object detection, complex backgrounds, and scale variance. The integration of the Area-Attention C2f (A2C2F) module significantly enhanced spatial feature representation using area-based attention and lightweight MLP blocks. Additionally, the use of Concat, Upsample, and stacked A2C2F blocks facilitated robust multi-scale feature fusion, preserving fine spatial details critical for detecting small and occluded objects. A novel decoupled detection head with lightweight attention-guided fusion further improved both precision and speed. According to experimental data, the suggested model performed better than the state-of-the-art techniques currently in use, achieving a mAP@50 of 58.8% and mAP@0.5:0.95 of 40.9%, clearly indicating its effectiveness for real-time UAV-based object detection scenarios.

## Supplementary Information

Below is the link to the electronic supplementary material.

Supplementary Material 1

Supplementary Material 2

Supplementary Material 3

## Data Availability

The datasets generated and/or analyzed during the current study are available in the Ultralytics repository at the following link: https://docs.ultralytics.com/datasets/detect/visdrone/All relevant data supporting the findings of this study can be accessed and utilized in accordance with the repository’s usage guidelines.
