# Supplementary material for: An efficient YOLOv12-based framework for detecting extremely small-scale objects
Source: Sci Rep. 2025 Dec 12;16:2062. doi: 10.1038/s41598-025-31803-7 (PMC12808633; doi:10.1038/s41598-025-31803-7)
Supplement: Supplementary file 1 — Supplementary Material 1 [file 41598_2025_31803_MOESM1_ESM.docx]

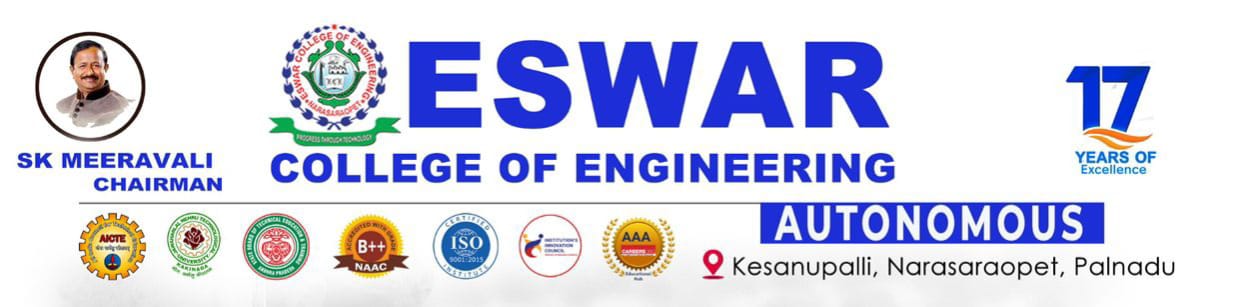

Date: 09-10 -2025

To
The Editorial Team,
Scientific Reports - International Journal
Publishers: Springer Nature.

Sub: Request for APC Waiver for Submitted Article - Reg.

Dear Sir,

I am Ponduri Vasanthi, corresponding author of the article titled “An Efficient YOLOv12-Based Framework for Detecting Extremely Small-Scale Objects”, which has been submitted to Scientific Reports. As a Assistant Professor, I do not have research funding, and my co-authors also do not have any financial support available for this work. Additionally, our institution has not provided any funding for the publication of this article. Given these circumstances, I would like to formally request a waiver for the Article Processing Charges (APC). I appreciate your consideration of this request and would be grateful for any support you can provide to facilitate the publication of our research. Please let me know if any further documentation is required.

Thank you for your time and assistance. I look forward to your response.

Authors:
1. A Chandrashekhar

Department of Mechanical Engineering, Faculty of Science and Technology, Icfai Foundation for Higher Education, Hyderabad, Telangana, India. 501203.

2. B Satyanarayana

Department of ECE, Professor, SASI institute of Technology and Engineering, Tadepalligudem.

3. Rajani Reddy Gorrepati

Department of Computer Science and Engineering, Koneru Lakshmaiah Education Foundation, Vaddeswaram, Andhra Pradesh, India.

4. Ponduri Vasanthi (Corresponding Author)

Department of Electronics and Communication Engineering, Eswar College of Engineering, Narasaraopet, Guntur, Andhra Pradesh, 522601, India

5. Kothala Lakshmi Prasanna
Department of Electronics and Communication Engineering, ACE Engineering College, Hyderabad

Thank you, Sir
Institution mail id: hoddet@eswarcollegeofengg.org

Dr. P. Vasanthi

DET HOD
