# Supplementary material for: An efficient YOLOv12-based framework for detecting extremely small-scale objects
Source: Sci Rep. 2025 Dec 12;16:2062. doi: 10.1038/s41598-025-31803-7 (PMC12808633; doi:10.1038/s41598-025-31803-7)
Supplement: Supplementary file 2 — Supplementary Material 2 [file 41598_2025_31803_MOESM2_ESM.doc]

**Data Availability Statement**

The datasets generated and/or analyzed during the current study are available in the Ultralytics repository at the following link:

https://docs.ultralytics.com/datasets/detect/visdrone/

All relevant data supporting the findings of this study can be accessed and utilized in accordance with the repository’s usage guidelines.
