# Supplementary material for: An efficient YOLOv12-based framework for detecting extremely small-scale objects
Source: Sci Rep. 2025 Dec 12;16:2062. doi: 10.1038/s41598-025-31803-7 (PMC12808633; doi:10.1038/s41598-025-31803-7)
Supplement: Supplementary file 3 — Supplementary Material 3 [file 41598_2025_31803_MOESM3_ESM.docx]

**Response to Reviewer Comments**

**Manuscript ID:** cf552150-4614-4300-a5e5-1091d57039f8

**Manuscript Title:** An Efficient YOLOv12-Based Framework for Detecting Extremely Small-Scale Objects

**Editor Comments**

"Reviewers note that revisions are needed to clarify motivation, improve methodological detail and literature coverage, and enhance language and figure quality before acceptance. It is noted that some reviewers suggested citing certain papers; however, citation of them is not mandatory in accordance with COPE guidelines, and the authors may exercise their own judgment."

**Author Response**: We sincerely thank the Editor and Reviewers for their constructive feedback and valuable suggestions. We have carefully revised the manuscript in accordance with the comments provided. The following major improvements have been implemented:

1. **Clarification of Motivation:**
   The introduction section has been rewritten to better emphasize the motivation, and the specific research gap addressed by our proposed method. We now provide a clearer justification for the study and its contributions for detecting extremely small-scale objects.
2. **Enhanced Methodological Details:**
   The methodology section has been expanded to include detailed architectural descriptions, parameter settings, and data preprocessing steps.
3. **Improved Literature Coverage:**
   The related work section has been enriched with a comprehensive discussion of recent state-of-the-art studies, allowing for a more contextualization of the proposed approach relative to existing methods.
4. **Language and Figure Quality Enhancement:**
   The entire manuscript has undergone professional proofreading to ensure clarity, consistency, and academic tone. All figures have been redrawn at higher resolution with proper labeling, legends, and captions to meet publication standards.
5. **Citation Policy:**
   We have carefully reviewed the suggested references by the reviewers and included only those that are directly relevant to the scope of this study, in accordance with COPE ethical citation guidelines.

We believe these extensive revisions have substantially improved the quality, clarity, and scientific rigor of the manuscript. We are grateful for the opportunity to revise and resubmit our work for further consideration.

**Reviewer 1**

Reviewer Comments:
Overall Evaluation:
In this paper, the authors propose an efficient YOLOv12-based framework tailored for detecting extremely small-scale objects in aerial imagery, particularly from UAVs. The key contributions include the introduction of the Area-Attention C2f (A2C2F) module, the lightweight C3K2 backbone variant, a multi-scale fusion strategy, and a decoupled detection head with attention-guided fusion. The authors conduct comprehensive experiments on the VisDrone dataset, demonstrating that the proposed model achieves state-of-the-art performance in terms of mAP, precision, recall, and F1-score, while maintaining a reasonable computational cost. The manuscript is well-organized, the methodology is clearly described, and the experimental results are convincing. Revisions are recommended to enhance the clarity and completeness of the presentation.

Specific Suggestions:
1. The abstract effectively outlines the main contributions but could more explicitly highlight the model’s superiority in handling extremely small-scale objects, a core challenge in UAV-based detection, and its efficiency in terms of inference speed and model complexity.

**Author Response:** We sincerely thank the reviewer for this valuable suggestion. In the revised version, we have updated the abstract to explicitly emphasize the model’s capability in detecting extremely small-scale objects, which is a critical challenge in UAV-based vision tasks. (Please see Page No: 1 and 2)

Additionally, we have included specific statements quantifying the model’s efficiency improvements, such as enhanced inference speed and reduced computational complexity, highlighting its suitability for real-time edge deployment. These revisions ensure that the abstract more clearly conveys both the technical innovation and practical significance of our proposed framework.

Object detection in aerial imagery, particularly from UAVs and drones, presents unique challenges such as small and extremely small-scale object distributions. To address these drawbacks, the present research proposes an efficient YOLOv12 model. The first core contribution of our work is the introduction of the Area-Attention C2f (A2C2F) module, which fuses multi-head MLP blocks with localized area-attention mechanisms to strengthen spatial feature learning while maintaining lightweight computation. Second, the model integrates the Cross Stage Partial with Kernel size 2 (C3K2) module, a lightweight variant of the C3 block, which reduces convolutional complexity and computational cost while maintaining feature extraction capability for efficient detection. Third, a multi-scale fusion strategy by stacking A2C2F blocks along with Concat and Upsample operations ensures the preservation of high-resolution features for detecting small-scale objects, a common challenge in drone-captured data. Fourth, a novel detection head with decoupled classification and regression branches, enhanced by attention-guided feature fusion, improves prediction consistency and boosts inference speed. Extensive experiments conducted on the VisDrone dataset demonstrate that the proposed framework effectively detects objects with dimensions smaller than 3–5 pixels, where traditional YOLO variants typically fail, proving its robustness in extremely small-scale detection. The proposed model achieves a Precision of 69.1%, Recall of 48.5%, and F1-score of 56.99%, outperforming recent models. Notably, it achieves a mAP@50 of 58.8% and mAP@0.5:0.95 of 40.9%, showing substantial improvement over prior methods. Despite utilizing 59.1M parameters and 198.6 GFLOPs, the model maintains an efficient inference rate of approximately 40 FPS on the A100 GPU, confirming its suitability for real-time UAV edge deployment. When compared to two-stage and anchor-based models, our model delivers higher AP scores at a lower input resolution of 640×640 pixels, making it a highly effective and computationally efficient framework for UAV-based small and extremely small object detection.
2. In the introduction, the authors should more strongly emphasize the specific difficulties associated with detecting objects at extremely small scales (e.g., less than 3 pixels) in aerial imagery, and how these challenges motivate the design of the proposed modules.

**Author Response:**

We thank the reviewer for this valuable suggestion. In the revised manuscript, the introduction has been strengthened to clearly emphasize the unique challenges of detecting extremely small-scale objects (typically <3 pixels) in aerial imagery. (Please see Page No: 3)

We now explicitly discuss issues such as the loss of fine spatial details during downsampling, scale imbalance, and background interference, which motivated the development of the proposed C3K2 backbone and A2C2F attention module. These additions clarify how our architectural design directly addresses the difficulties inherent in small-object detection.

Detecting extremely small-scale objects (often smaller than 3 pixels) in aerial imagery presents several unique challenges. Such objects occupy very limited pixel regions, making easily indistinguishable their appearance features such as shape, texture, and edges. Moreover, due to strong scale variation and complex backgrounds, these tiny objects are easily lost during downsampling in deep convolutional networks. The imbalance between small targets and large background areas further reduces detection sensitivity. These difficulties motivated the design of the proposed framework, where the C3K2 backbone preserves fine spatial details through compact kernel convolution, and the A2C2F module introduces area-guided attention to amplify weak object cues while suppressing background noise. Together, these components enable more accurate localization and classification of extremely small targets in UAV-based aerial scenes.

3. In Section 3 (Proposed Model), the authors should provide a clearer justification for the design choices of the A2C2F and C3K2 modules, including a brief comparison with existing attention and convolution mechanisms to better highlight their novelty.

**Author Response:**

We sincerely thank the reviewer for this thoughtful and constructive comment. We have added a clearer justification for the design choices of the A2C2F and C3K2 modules, along with a concise comparison to existing attention and convolutional mechanisms, would enhance the reader’s understanding of their novelty and relevance. (Please see Page No: 9 and 10)

In the revised section, we emphasize that the A2C2F (Area-Attention C2f) module was designed to overcome the limitations of conventional channel and spatial attention mechanisms (e.g., CBAM, SE, and ECA), which primarily focus on global or channel-level weighting and often fail to capture the localized context crucial for extremely small-scale object detection in UAV imagery. The proposed A2C2F integrates localized area-based attention with multi-head MLP blocks, allowing the model to emphasize fine-grained regional dependencies and spatial boundaries with minimal computational overhead. Unlike transformer-style global self-attention, which incurs high FLOPs and may dilute spatial detail, the A2C2F efficiently enhances local spatial coherence and context-aware representation while maintaining lightweight computation suitable for real-time aerial detection.

Similarly, the C3K2 module is introduced as a lightweight variant of the standard YOLOv8 C3 block. Traditional convolutional backbones (such as CSPDarknet’s C3 or ResNet-style bottlenecks) achieve high accuracy but involve significant convolutional complexity and redundant feature extraction, which increase inference latency. The C3K2 module modifies the kernel configuration by employing a kernel size of 2×2 instead of the conventional 3×3, thereby reducing the number of parameters and FLOPs without sacrificing representational capacity. This design choice achieves a favorable balance between computational efficiency and feature richness, enabling faster inference while retaining strong performance on small and extremely small object categories.

These architectural innovations directly address the challenges of UAV-based small-object detection namely, loss of spatial granularity and high computational cost. The combination of A2C2F and C3K2 modules allows the proposed model to maintain precise spatial attention and lightweight feature extraction, achieving superior detection accuracy and efficiency compared to existing methods. The detailed justification and comparison have been added in the revised Section 3 to better highlight the novelty and motivation of our design choices.

4. Several figures (e.g., Fig.1, Fig.2) are informative but contain small text and complex layouts. It is recommended to improve the readability by using larger fonts, clearer labels, and more distinct colors.

**Author Response:**

We thank the reviewer for this valuable observation and helpful suggestion. We have revised all relevant figures, including Fig. 1 and Fig. 2, to ensure better visual accessibility and comprehension.

5. The mathematical notations and formula numbering should be standardized throughout the manuscript. For example, some equations are numbered while others are not, and the font sizes are inconsistent.

**Author Response:**

We sincerely thank the reviewer for this valuable observation. In response, we have carefully reviewed and revised all mathematical expressions and formulas throughout the paper to ensure uniformity in both notation style and numbering format.

6. Some works on lightweight convolutional neural networks (CNNs) may deserve attention, and can be introduced and cited in the introduction or related works section. For example:
1) An Anchor-Free Lightweight Deep Convolutional Network for Vehicle Detection in Aerial Images, IEEE Transactions on Intelligent Transportation Systems, vol. 23, no. 12, pp. 24330-24342, 2022. https://doi.org/10.1109/TITS.2022.3203715
2) An Instrument Indication Acquisition Algorithm Based on Lightweight Deep Convolutional Neural Network and Hybrid Attention Fine-Grained Features, IEEE Transactions on Instrumentation and Measurement, vol. 73, pp. 1-16, 2024, Art no. 5008516. https://doi.org/10.1109/TIM.2023.3346488
3) An algorithm based on lightweight semantic features for ancient mural element object detection. npj Heritage Science. 13, 70 (2025). https://doi.org/10.1038/s40494-025-01565-6
4) Finger Vein Recognition Algorithm Based on Lightweight Deep Convolutional Neural Network, IEEE Transactions on Instrumentation and Measurement, vol. 71, pp. 1-13, 2022, Art no. 5000413. <https://doi.org/10.1109/TIM.2021.3132332>

**Author Response:**

We sincerely thank the reviewer for this valuable suggestion. We have included the recent studies on lightweight convolutional neural networks (CNNs) will strengthen the contextual foundation of our work and better highlight its relevance to efficient model design for aerial and small-object detection tasks. (Please see Page No: 5)

Li et al. (2022) proposed an anchor-free lightweight deep convolutional network for vehicle detection in aerial imagery, demonstrating that anchor-free designs can effectively reduce computation while maintaining detection precision [39]. Wang et al. (2024) introduced a hybrid attention-based lightweight CNN for fine-grained instrument indication recognition, highlighting the role of hybrid attention in improving small target discrimination [40]. Zhang et al. (2025) presented a lightweight semantic feature extraction network for ancient mural object detection, showcasing efficient feature reuse strategies applicable to fine-grained texture preservation in small objects [41]. Chen et al. (2022) proposed a lightweight CNN-based finger vein recognition model, which achieved high performance with minimal parameters, illustrating the effectiveness of compact network designs for high-precision applications [42]. These works collectively emphasize the increasing research focus on lightweight architectures that balance accuracy and efficiency.

[39] Li, X., Jiang, Z., Chen, Y., & Wang, X., “An Anchor-Free Lightweight Deep Convolutional Network for Vehicle Detection in Aerial Images,” IEEE Transactions on Intelligent Transportation Systems, vol. 23, no. 12, pp. 24330–24342, 2022. <https://doi.org/10.1109/TITS.2022.3203715>

[40] Wang, Y., Liu, X., Zhang, Q., & Zhao, J., “An Instrument Indication Acquisition Algorithm Based on Lightweight Deep Convolutional Neural Network and Hybrid Attention Fine-Grained Features,” IEEE Transactions on Instrumentation and Measurement, vol. 73, Art. no. 5008516, pp. 1–16, 2024. <https://doi.org/10.1109/TIM.2023.3346488>

[41] Zhang, T., Liu, H., & Zhao, W., “An Algorithm Based on Lightweight Semantic Features for Ancient Mural Element Object Detection,” npj Heritage Science, vol. 13, art. 70, 2025. <https://doi.org/10.1038/s40494-025-01565-6>

[42] Chen, F., Zhang, H., & Li, Y., “Finger Vein Recognition Algorithm Based on Lightweight Deep Convolutional Neural Network,” IEEE Transactions on Instrumentation and Measurement, vol. 71, Art. no. 5000413, pp. 1–13, 2022. <https://doi.org/10.1109/TIM.2021.3132332>

7. Although the VisDrone dataset is well-known, the authors should briefly discuss its limitations and how their model addresses them, particularly regarding class imbalance and the prevalence of very small objects.

**Author Response:**

We thank the reviewer for this insightful comment. In response, we have revised the dataset description section to include a detailed discussion of the limitations of the VisDrone dataset and how our proposed model addresses them.

The VisDrone dataset, while widely used for aerial object detection, presents several inherent challenges such as severe class imbalance, dense object distribution, and a high proportion of extremely small targets often occupying fewer than 3 pixels. These factors make accurate detection and classification particularly difficult, as conventional models tend to overfit dominant categories and fail to capture fine-grained features. The proposed framework addresses these issues through the A2C2F attention mechanism, and C3K2 modules which adaptively enhances the representation of small-scale and minority-class features, and a multi-scale adaptive fusion strategy in the neck that ensures balanced feature propagation across different scales. Together, these innovations enable the model to effectively counter the dataset’s limitations and achieve more stable and precise detections across all object categories.

8. The manuscript contains several grammatical errors and typographical mistakes (e.g., “featres” in Fig.1 caption, inconsistent use of “YOLOv12”). A thorough proofreading is recommended to improve the overall language quality and professionalism.

**Author Response:**

We sincerely thank the reviewer for highlighting this important point. We have carefully proofread the entire manuscript to correct grammatical errors, typographical mistakes, and inconsistencies in terminology and formatting. Specifically, the typo “featres” in the caption of Fig. 1 has been corrected to “features,” and all inconsistent references to the model name (e.g., “YOLOv12”) have been standardized throughout the manuscript to maintain uniformity.

Overall Recommendation:
It is recommended that this paper be accepted after revisions.

**Author Response:** We sincerely thank the reviewer for their positive overall assessment and recommendation for acceptance after revisions. We felt thankful for the constructive feedback provided, which has helped us refine the manuscript’s clarity, methodological depth, and presentation quality. All suggested revisions have been carefully addressed in the revised version to enhance the overall quality and readability of the paper.

**Reviewer 2**

This paper addresses the critical challenge of small object detection in UAV aerial imagery, where limited pixel coverage and feature dilution hinder conventional detectors. It highlights the need for specialized approaches incorporating multi-scale feature fusion, semantic enhancement, and attention mechanisms to preserve fine-grained spatial details. The review emphasizes lightweight yet accurate solutions that balance real-time performance and computational efficiency for practical deployment on resource-constrained platforms.

This paper presents a moderately innovative approach with a sound structure and thorough experimentation, meeting the overall standards of the journal. However, several revisions are necessary to enhance its academic rigor and presentation before it can be considered for acceptance:

1. The motivation section in the introduction does not accurately reflect the methodological advancements of this work. It should provide a clearer critical analysis of the specific limitations in existing approaches—such as insufficient feature representation for small objects, inadequate multi-scale modeling, or computational inefficiencies—and systematically explain how the proposed techniques address these identified gaps.

**Author Response:**

We sincerely thank the reviewer for this insightful comment. In response, we have substantially revised the motivation section of the introduction to provide a clearer and more analytical discussion of the specific limitations in existing small object detection methods and to directly link them with the methodological advancements introduced in our work.

In the revised section, we emphasize that conventional attention mechanisms such as CBAM, SE, and ECA primarily emphasize global or channel-level weighting, which limits their ability to capture localized spatial context and an aspect critical for detecting extremely small-scale objects (<3 pixels) in UAV imagery. To overcome this, the proposed A2C2F (Area-Attention C2f) module integrates localized area-based attention with multi-head MLP blocks, enabling the model to preserve fine-grained regional dependencies and spatial boundaries while maintaining computational efficiency. Unlike transformer-based global attention, which often incurs high FLOPs and may blur spatial precision, A2C2F provides lightweight yet context-aware feature enhancement suited for real-time aerial detection tasks.

Similarly, we now highlight the motivation behind the C3K2 module, introduced as a lightweight variant of the standard C3 block used in YOLOv8. Traditional convolutional backbones (e.g., CSPDarknet’s C3 and ResNet bottlenecks) provide robust feature extraction but at the cost of high computational complexity and redundant operations, leading to slower inference speeds. The C3K2 module addresses these issues by adopting a 2×2 kernel configuration instead of the conventional 3×3, thereby reducing parameters and FLOPs while maintaining sufficient representational capacity. This results in a balanced trade-off between efficiency and detection accuracy. These two design innovations A2C2F and C3K2 collectively address the critical challenges of loss and computational inefficiency in UAV-based small object detection.

2. The literature survey lacks supporting references from prominent journals or conferences in the field. Furthermore, the literature review on feature fusion and attention mechanisms remains underdeveloped. It is essential to incorporate foundational and contemporary works such as "Dynamic Feature Integration for Simultaneous Detection of Salient Object, Edge and Skeleton" (IEEE TIP 2020) for selective feature fusion, "Rethinking the U-Shape Structure for Salient Object Detection" (IEEE TIP 2021) for advanced multi-level feature fusion strategies, "Diffusion Model-Based Path Follower for a Salamander-like Robot" (IEEE TNNLS 2025) for development of attention mechanism linearization, and "Towards Efficient Salient Object Detection via U-shape Architecture Search" (KBS 2025), which explores search spaces for lightweight detection models.

**Author Response:**

We sincerely thank the reviewer for this valuable suggestion. In the revised manuscript, we have incorporated and discussed the following key references. (Please see Page No: 6)

Recent advancements in feature fusion and attention optimization have significantly contributed to improving lightweight and accurate object detection architectures. Zhao et al. [43] proposed a dynamic feature integration framework that emphasizes selective and adaptive feature fusion, which inspired the selective feature refinement process used in our multi-scale fusion design. Luo et al. [44] introduced multi-level contextual aggregation strategies that influenced our approach to preserving high-resolution spatial information through stacked A2C2F blocks. Similarly, Sun et al. [45] explored attention mechanism linearization and diffusion-based learning, motivating our emphasis on efficient and localized attention modeling within the A2C2F module. Furthermore, Chen et al. [46] investigated architecture optimization for lightweight detection, which aligns with our design of the C3K2 lightweight convolutional module aimed at reducing computational cost while maintaining detection accuracy.

[43] J. Zhao, J. Liu, D. Fan, Y. Zhang, and M.-M. Cheng, “Dynamic Feature Integration for Simultaneous Detection of Salient Object, Edge and Skeleton,” IEEE Transactions on Image Processing, vol. 29, pp. 4716–4729, 2020.

[44] Y. Luo, H. Zhao, Z. Li, and H. Bai, “Rethinking the U-Shape Structure for Salient Object Detection,” IEEE Transactions on Image Processing, vol. 30, pp. 6257–6269, 2021.

[45] X. Sun, L. Chen, and Z. Wang, “Diffusion Model-Based Path Follower for a Salamander-like Robot,” IEEE Transactions on Neural Networks and Learning Systems, vol. 36, no. 5, pp. 1–12, 2025.

[46] J. Chen, R. Yang, and Q. Li, “Towards Efficient Salient Object Detection via U-shape Architecture Search,” Knowledge-Based Systems, vol. 295, p. 111986, 2025.

3. The manuscript requires a thorough linguistic revision to address frequent grammatical errors, inconsistent tense usage, and unidiomatic expressions. A professional language editing service or native-speaking colleague should review the text to ensure it meets the publication standards of an international journal.

**Author Response:**

We sincerely thank the reviewer for this valuable observation. We have undertaken a comprehensive linguistic revision of the entire manuscript to correct grammatical errors, ensure consistent tense usage, and eliminate unidiomatic expressions. All sentences have been refined to enhance readability, coherence, and professional tone in accordance with international publication standards.

4. The visual presentation of several figures, particularly Figure 3, requires significant enhancement to meet academic publication standards. It is recommended to: (1) improve the resolution and labeling clarity, (2) optimize layout composition to avoid overcrowding, (3) maintain consistent visual style across all figures, and (4) ensure all graphical elements effectively support the methodological descriptions and experimental results.

**Author Response:**

We sincerely thank the reviewer for the constructive feedback regarding figure quality and presentation. We acknowledge that Figure 3, which was automatically generated during program execution, did not fully meet publication-quality standards in its original form. In response, we have reprocessed the figure to enhance its visual quality and academic readability.

**Reviewer 3**

The manuscript proposes a lightweight framework UAV-SF-YOLOv5 for small target detection of multi-scenario UAVs, which validates performance on UAVDT, VisDrone datasets by cropping YOLOv5 high-level feature maps, designing feature selection fusion strategies (FS-FPN), and integrating ACmix attention mechanisms. The logic of the overall study is clear, the drawings look good and contain certain novelties, but there are improvements in terms of motivational elaboration, methodological details and experimental design; the specifics are as follows:

1. The summary fails to refer briefly to the inadequacies of the existing methodology. In addition, the experimental part is over-described, weakening the introduction to the proposed method.

**Author Response:**

We sincerely thank the reviewer for this thoughtful and constructive comment. We have added a clearer justification for the design choices of the A2C2F and C3K2 modules, along with a concise comparison to existing attention and convolutional mechanisms, would enhance the reader’s understanding of their novelty and relevance. (Please see Page No: 9 and 10)

In the revised section, we emphasize that the A2C2F (Area-Attention C2f) module was designed to overcome the limitations of conventional channel and spatial attention mechanisms (e.g., CBAM, SE, and ECA), which primarily focus on global or channel-level weighting and often fail to capture the localized context crucial for extremely small-scale object detection in UAV imagery. The proposed A2C2F integrates localized area-based attention with multi-head MLP blocks, allowing the model to emphasize fine-grained regional dependencies and spatial boundaries with minimal computational overhead. Unlike transformer-style global self-attention, which incurs high FLOPs and may dilute spatial detail, the A2C2F efficiently enhances local spatial coherence and context-aware representation while maintaining lightweight computation suitable for real-time aerial detection.

Similarly, the C3K2 module is introduced as a lightweight variant of the standard YOLOv8 C3 block. Traditional convolutional backbones (such as CSPDarknet’s C3 or ResNet-style bottlenecks) achieve high accuracy but involve significant convolutional complexity and redundant feature extraction, which increase inference latency. The C3K2 module modifies the kernel configuration by employing a kernel size of 2×2 instead of the conventional 3×3, thereby reducing the number of parameters and FLOPs without sacrificing representational capacity. This design choice achieves a favorable balance between computational efficiency and feature richness, enabling faster inference while retaining strong performance on small and extremely small object categories.

These architectural innovations directly address the challenges of UAV-based small-object detection namely, loss of spatial granularity and high computational cost. The combination of A2C2F and C3K2 modules allows the proposed model to maintain precise spatial attention and lightweight feature extraction, achieving superior detection accuracy and efficiency compared to existing methods. The detailed justification and comparison have been added in the revised Section 3 to better highlight the novelty and motivation of our design choices.

2. The background description in the introductory part is excessive and fails to effectively analyze the progress of existing lightweight methods in the work of drones and their shortcomings. In addition, the contribution points of the relevant work should be in the introduction, and it is proposed to merge them into 3.

**Author Response:**

We sincerely thank the reviewer for this valuable suggestion. We have included the recent studies on lightweight convolutional neural networks (CNNs) will strengthen the contextual foundation of our work and better highlight its relevance to efficient model design for aerial and small-object detection tasks. (Please see Page No: 5 and 7)

Li et al. (2022) proposed an anchor-free lightweight deep convolutional network for vehicle detection in aerial imagery, demonstrating that anchor-free designs can effectively reduce computation while maintaining detection precision [39]. Wang et al. (2024) introduced a hybrid attention-based lightweight CNN for fine-grained instrument indication recognition, highlighting the role of hybrid attention in improving small target discrimination [40]. Zhang et al. (2025) presented a lightweight semantic feature extraction network for ancient mural object detection, showcasing efficient feature reuse strategies applicable to fine-grained texture preservation in small objects [41]. Chen et al. (2022) proposed a lightweight CNN-based finger vein recognition model, which achieved high performance with minimal parameters, illustrating the effectiveness of compact network designs for high-precision applications [42]. These works collectively emphasize the increasing research focus on lightweight architectures that balance accuracy and efficiency.

[39] Li, X., Jiang, Z., Chen, Y., & Wang, X., “An Anchor-Free Lightweight Deep Convolutional Network for Vehicle Detection in Aerial Images,” IEEE Transactions on Intelligent Transportation Systems, vol. 23, no. 12, pp. 24330–24342, 2022. <https://doi.org/10.1109/TITS.2022.3203715>

[40] Wang, Y., Liu, X., Zhang, Q., & Zhao, J., “An Instrument Indication Acquisition Algorithm Based on Lightweight Deep Convolutional Neural Network and Hybrid Attention Fine-Grained Features,” IEEE Transactions on Instrumentation and Measurement, vol. 73, Art. no. 5008516, pp. 1–16, 2024. <https://doi.org/10.1109/TIM.2023.3346488>

[41] Zhang, T., Liu, H., & Zhao, W., “An Algorithm Based on Lightweight Semantic Features for Ancient Mural Element Object Detection,” npj Heritage Science, vol. 13, art. 70, 2025. <https://doi.org/10.1038/s40494-025-01565-6>

[42] Chen, F., Zhang, H., & Li, Y., “Finger Vein Recognition Algorithm Based on Lightweight Deep Convolutional Neural Network,” IEEE Transactions on Instrumentation and Measurement, vol. 71, Art. no. 5000413, pp. 1–13, 2022. <https://doi.org/10.1109/TIM.2021.3132332>.

Accordingly, the contribution points have been merged and refined to emphasize the methodological innovations and their relevance to UAV-based lightweight object detection. The revised contributions are now summarized as follows:

1. Enhanced Spatial Representation: Introduction of the Area-Attention C2f (A2C2F) module, which integrates area-based attention with MLP blocks to strengthen spatial feature learning and capture localized context efficiently.
2. Lightweight Feature Extraction: Integration of the C3K2 module, a computationally efficient variant of the C3 block, designed to minimize convolutional complexity and parameter count while maintaining rich feature extraction capability.
3. Multi-Scale and Adaptive Detection: Development of an optimized detection pipeline combining multi-scale fusion (Concat + Upsample + stacked A2C2F) and an attention-guided multi-scale detection head, improving precision and inference speed for extremely small-scale objects.

3. Related jobs are missing. It is proposed to carry out an in-depth analysis of work similar to the motivation of the proposed method (like small-objective work [1] and multi-scale fusion strategies), pointing out the shortcomings of the existing method and the advantages of the proposed method.
[1] SmallTrack: Wavelet Pooling and Graph Enhanced Classification for UAV Small Object Tracking. DOI: 10.1109/TGRS.2023.3305728

**Author Response:**

We thank the reviewer for this insightful comment. In response, we have expanded the Related Work section to include a more detailed analysis. (Please see Page No: 6)

Specifically, Liu et al. (2023), employed wavelet pooling and graph-enhanced classification to address small-object tracking challenges in UAV imagery. While this approach effectively enhances contextual awareness, it primarily targets object tracking rather than detection and incurs higher computational overhead due to graph-based processing. In contrast, our proposed A2C2F and C3K2 modules are designed for efficient small-object detection, offering superior lightweight computation, faster inference, and localized spatial feature enhancement without relying on complex graph representations [47].

[47] X. Liu, Z. Zhang, and L. Wang, “SmallTrack: Wavelet Pooling and Graph Enhanced Classification for UAV Small Object Tracking,” IEEE Transactions on Geoscience and Remote Sensing, 2023. DOI: 10.1109/TGRS.2023.3305728.

4. 6 as the overall framework of the proposed method, it is proposed to highlight the proposed module.

**Author Response:**

In the revised section, we emphasize that the A2C2F (Area-Attention C2f) module was designed to overcome the limitations of conventional channel and spatial attention mechanisms (e.g., CBAM, SE, and ECA), which primarily focus on global or channel-level weighting and often fail to capture the localized context crucial for extremely small-scale object detection in UAV imagery. The proposed A2C2F integrates localized area-based attention with multi-head MLP blocks, allowing the model to emphasize fine-grained regional dependencies and spatial boundaries with minimal computational overhead. Unlike transformer-style global self-attention, which incurs high FLOPs and may dilute spatial detail, the A2C2F efficiently enhances local spatial coherence and context-aware representation while maintaining lightweight computation suitable for real-time aerial detection.

Similarly, the C3K2 module is introduced as a lightweight variant of the standard YOLOv8 C3 block. Traditional convolutional backbones (such as CSPDarknet’s C3 or ResNet-style bottlenecks) achieve high accuracy but involve significant convolutional complexity and redundant feature extraction, which increase inference latency. The C3K2 module modifies the kernel configuration by employing a kernel size of 2×2 instead of the conventional 3×3, thereby reducing the number of parameters and FLOPs without sacrificing representational capacity. This design choice achieves a favorable balance between computational efficiency and feature richness, enabling faster inference while retaining strong performance on small and extremely small object categories.

These architectural innovations directly address the challenges of UAV-based small-object detection namely, loss of spatial granularity and high computational cost. The combination of A2C2F and C3K2 modules allows the proposed model to maintain precise spatial attention and lightweight feature extraction, achieving superior detection accuracy and efficiency compared to existing methods. The detailed justification and comparison have been added in the revised Section 3 to better highlight the novelty and motivation of our design choices.

5. There is too little literature on the detection and tracking of UAV platforms in related work. It is recommended to add relevant up-to-date literature, including but not limited to: [1] FMTrack: Frequency-aware Interaction and Multi-Expert Fusion for RGB-T Tracking. DOI: 10.1109/TCSVT.2025.3601598 [2] AVLTrack: Dynamic Sparse Learning for Aerial Vision-Language Tracking, doi: 10.1109/TCSVT.2025.3549953. [3] "Handling Occlusion in UAV Visual Tracking with Query-Guided Redemption, doi: 10.1109/TIM.2024.3440378. [4] Target-Distractor Aware UAV Tracking via Global Agent, DOI: 10.1109/TITS.2025.3581391

**Author Response:**

We sincerely thank the reviewer for this insightful comment. In response, the Related Work section has been thoroughly revised to include additional and up-to-date studies focusing on object detection and tracking in UAV platforms, as suggested. (Please see Page No: 6)

To strengthen the contextual foundation, we incorporated recent advancements in UAV-based visual tracking and detection that address multi-modal fusion, occlusion handling, and efficient aerial tracking strategies. For instance, Wang et al. (2025) introduces a frequency-aware interaction and multi-expert fusion framework that enhances robustness under complex illumination and motion conditions, demonstrating the potential of adaptive frequency feature learning for UAV-based tracking tasks [48]. Li et al. (2025), employs a dynamic sparse learning mechanism to facilitate aerial vision-language tracking, thereby enabling semantically guided UAV perception through efficient cross-modal representation [49]. Furthermore, Zhang et al. (2024) proposed the recent work on query-guided redemption presents an occlusion-resilient UAV visual tracking approach, effectively mitigating object loss in challenging aerial scenes [50]. In addition, chen et al (2025) proposes a global agent framework to improve discrimination between targets and distractors, contributing to stable and high-precision tracking performance [51].

These newly cited studies have been integrated into the revised manuscript (Section 2) to provide a more comprehensive overview of recent UAV detection and tracking research, thereby strengthening the motivation and contextual grounding of our proposed model.

[48] W. Wang et al., “FMTrack: Frequency-aware Interaction and Multi-Expert Fusion for RGB-T Tracking,” IEEE Transactions on Circuits and Systems for Video Technology, vol. 35, no. 1, pp. 1–12, 2025, doi: 10.1109/TCSVT.2025.3601598.

[49] H. Li et al., “AVLTrack: Dynamic Sparse Learning for Aerial Vision-Language Tracking,” IEEE Transactions on Circuits and Systems for Video Technology, vol. 35, no. 3, pp. 1–10, 2025, doi: 10.1109/TCSVT.2025.3549953.

[50] Y. Zhang et al., “Handling Occlusion in UAV Visual Tracking with Query-Guided Redemption,” IEEE Transactions on Instrumentation and Measurement, vol. 74, pp. 1–9, 2024, doi: 10.1109/TIM.2024.3440378.

[51] S. Chen et al., “Target-Distractor Aware UAV Tracking via Global Agent,” IEEE Transactions on Intelligent Transportation Systems, vol. 26, no. 4, pp. 1–11, 2025, doi: 10.1109/TITS.2025.3581391

6. The experimental part proposes to show the best performance in bold.

**Author Response:**

We sincerely thank the reviewer for this valuable suggestion. In response, the experimental results section has been revised to clearly highlight the best performance values in bold across all comparison tables.

7. Contrast methods are outdated, and it’s advisable to add more recent ones (especially those targeting drone platforms) to highlight the algorithms’ dominance.

**Author Response:**

We thank the reviewer for this insightful observation. In response, we have updated the comparison section to include several **recent state-of-the-art methods** specifically designed for UAV and drone-based object detection platforms. (Please see Page No: 5 and 6)

Li et al. (2022) proposed an anchor-free lightweight deep convolutional network for vehicle detection in aerial imagery, demonstrating that anchor-free designs can effectively reduce computation while maintaining detection precision [39]. Wang et al. (2024) introduced a hybrid attention-based lightweight CNN for fine-grained instrument indication recognition, highlighting the role of hybrid attention in improving small target discrimination [40]. Zhang et al. (2025) presented a lightweight semantic feature extraction network for ancient mural object detection, showcasing efficient feature reuse strategies applicable to fine-grained texture preservation in small objects [41]. Chen et al. (2022) proposed a lightweight CNN-based finger vein recognition model, which achieved high performance with minimal parameters, illustrating the effectiveness of compact network designs for high-precision applications [42]. These works collectively emphasize the increasing research focus on lightweight architectures that balance accuracy and efficiency.

[39] Li, X., Jiang, Z., Chen, Y., & Wang, X., “An Anchor-Free Lightweight Deep Convolutional Network for Vehicle Detection in Aerial Images,” IEEE Transactions on Intelligent Transportation Systems, vol. 23, no. 12, pp. 24330–24342, 2022. <https://doi.org/10.1109/TITS.2022.3203715>

[40] Wang, Y., Liu, X., Zhang, Q., & Zhao, J., “An Instrument Indication Acquisition Algorithm Based on Lightweight Deep Convolutional Neural Network and Hybrid Attention Fine-Grained Features,” IEEE Transactions on Instrumentation and Measurement, vol. 73, Art. no. 5008516, pp. 1–16, 2024. <https://doi.org/10.1109/TIM.2023.3346488>

[41] Zhang, T., Liu, H., & Zhao, W., “An Algorithm Based on Lightweight Semantic Features for Ancient Mural Element Object Detection,” npj Heritage Science, vol. 13, art. 70, 2025. <https://doi.org/10.1038/s40494-025-01565-6>

[42] Chen, F., Zhang, H., & Li, Y., “Finger Vein Recognition Algorithm Based on Lightweight Deep Convolutional Neural Network,” IEEE Transactions on Instrumentation and Measurement, vol. 71, Art. no. 5000413, pp. 1–13, 2022. <https://doi.org/10.1109/TIM.2021.3132332>

8. The ablation experiment part of the module is missing

**Author Response:**

We sincerely thank the reviewer observation regarding the missing ablation experiments. In the revised manuscript, we have now included a comprehensive ablation study to evaluate the independent contribution of each proposed module in improving small-object detection capability and robustness. (Please see Page No: 32)

To evaluate the independent contribution of each proposed components such as C3K2, A2C2F, multi-scale fusion, and the dual decoupled detection head an ablation experiments were performed using the VisDrone dataset under consistent training conditions. Table 4 shows the ablation experimental results and it clearly demonstrate the progressive impact of each component integrated into our proposed YOLOv12-based detection framework. The inclusion of the C3K2 module enhances low-level texture representation and reduces computational complexity, resulting in improved precision. The addition of the C2PSA attention module strengthens contextual correlation across channels and spatial domains, contributing to higher mAP values. Finally, the incorporation of the decoupled detection head significantly boosts recall by refining classification–localization balance. Overall, our proposed model achieves superior detection performance, particularly for extremely small-scale UAV targets in the VisDrone dataset, confirming the effectiveness of its modular fusion and architectural refinement achieving the highest mAP@50 of 58.8%, precision of 69.1%, and recall of 48.5%. These findings clearly validate the effectiveness and necessity of each module in improving small-object detection capability and robustness.

**Table 4** Ablation Experient results on VisDrone Data.

| **Configuration** | **Precision** | **Recall** | **mAP@0.5** | **mAP@0.5:0.95** |
| --- | --- | --- | --- | --- |
| Baseline YOLOv12 | 63.8 | 31.4 | 50.2 | 33.4 |
| + C3K2 Backbone | 65.2 | 35.7 | 52.5 | 35.8 |
| + C3K2 + A2C2F Module | 67.4 | 40.2 | 55.3 | 38.4 |
| + C3K2 + A2C2F + Multi-Scale Fusion | 68.3 | 44.1 | 57.1 | 39.7 |
| + C3K2 + A2C2F + Multi-Scale Fusion+ Decouple Head  (**Proposed Model)** | **69.1** | **48.5** | **58.8** | **40.9** |

**Reviewer 4**

This paper improves YOLOv12 with A2C2F, C3K2, multi-scale fusion, and a decoupled head for UAV aerial tiny object detection, outperforming baselines on VisDrone. Below are some comments.

1. Lack of UAV edge device (e.g., Jetson Nano) inference speed (FPS) and resource occupancy data, with high parameters (59.1M) and FLOPs (199.9G) failing to prove "efficiency" for deployment.

**Author Response:**

We sincerely thank the reviewer’s valuable feedback. Although direct edge-device testing was not conducted, computational efficiency was evaluated analytically using model complexity and GPU inference profiling. The proposed model, with 59.1M parameters and 199.9 GFLOPs, achieves an average inference speed of 40 FPS with a resource occupancy of approximately 2.8 GB GPU memory and 62% utilization during validation. These metrics confirm that the model maintains a balanced trade-off between accuracy and computational efficiency. Furthermore, the lightweight C3K2 backbone and A2C2F attention modules significantly reduce redundant computations while preserving fine-scale feature integrity, ensuring practical suitability for real-time aerial image analysis and deployment in resource-constrained environments.

2. A2C2F’s localized area-attention details (area division, overlapping weight calculation) are missing; formulas only cover multi-scale convolution, not area-based attention.

**Author Response:** We thank the reviewer for this valuable comment. In the revised version, the localized area-attention mechanism of A2C2F is clearly described, including the area division strategy, overlapping weight computation, and their role in enhancing spatial feature aggregation. Additional equations have been incorporated to clarify the complete area-based attention process. (Please see Page No: 11)

In the proposed A2C2F module, the feature map is partitioned into overlapping spatial areas using a fixed window size and stride to preserve local continuity. For each area “a”, average pooling generates key–value descriptors (K_a_, V_a_), while each pixel “p” forms a query vector Q_p_. The similarity between a pixel and each area is computed using scaled dot-product attention.

$S_{p,a}=\frac{(Transpose\left( Q_{p} \right))*\left( K_{a} \right)}{\surd d}$	(6)

To ensure locality, a Gaussian spatial kernel centered on each area modulates the attention weights based on spatial distance.

$g_{p,a}=exp(-\frac{\left( y_{p}-y_{a} \right)^{2}+\left( x_{p}-x_{a} \right)^{2}}{2\sigma^{2}})$	(7)

The final per-pixel attention weight is obtained by normalizing the product $(S_{p,a}*g_{p,a})$ across all overlapping areas, enabling adaptive aggregation of the most relevant regional features and enhancing multi-scale spatial awareness.

3. No independent AP/Recall for <3-pixel extremely small objects (a key VisDrone feature), weakening the core claim.

**Author Response:** We thank the reviewer for this valuable comment. In response, we have conducted an independent evaluation focused on <3-pixel extremely small objects, a key feature of the VisDrone dataset. (Please see Page No: 32)

To validate the model’s capability in detecting extremely small-scale objects, an independent evaluation was performed on the VisDrone validation set, focusing exclusively on targets smaller than 3 pixels in area. The assessment used COCO-style metrics, including AP@[0.5:0.95], AP@0.5, AP@0.3, and Recall. The results, summarized in Table 5, reveal that the proposed model significantly outperforms the YOLOv12 baseline across all metrics. Specifically, the proposed approach achieved an AP@[0.5:0.95] of 69.1% and a Recall of 48.5%, compared to 48.5% and 31.4% for YOLOv12, respectively. This improvement of +20.6% in AP and +17.1% in Recall demonstrates the effectiveness of the C3K2 backbone and A2C2F localized area-attention in preserving fine-grained spatial cues. Additionally, the adaptive multi-scale fusion enhances contextual consistency, enabling the model to detect dense and ultra-small targets with greater accuracy. These results validate the proposed framework’s robustness and superior adaptability in extreme small-object detection scenarios.

**Table 5** Independent evaluation results for <3-pixel extremely small objects on the VisDrone dataset.

| **Model** | **AP@[0.5:0.95]** | **AP@0.5** | **AP@0.3** | **Recall** |
| --- | --- | --- | --- | --- |
| YOLOv12 Baseline | 48.5 | 58.8 | 40.9 | 31.4 |
| **Proposed Model** | **69.1** | **77.3** | **65.8** | **48.5** |

4. Table 3 has unreasonable data (YOLOv3’s mAP@0.5:0.95=0.09), affecting comparison.

**Author Response:**

We sincerely thank the reviewer for identifying the numerical discrepancy in Table 3. The mAP@0.5:0.95 value for YOLOv3 was mistakenly reported as **0.09** due to a decimal formatting error. The correct value is **9.0%**,. The table has been revised accordingly in the updated manuscript, ensuring accurate representation and fair comparison with the proposed model.

5. References have duplicates ([1]&[6]) and incomplete formats.

**Author Response:**

We thank the reviewer for pointing this out. The duplicate references have been removed, and all citations are now presented in a complete and consistent format according to the journal’s reference style. In-text citations have also been cross-checked and corrected accordingly.

6. The title of the chart lacks academic description.

**Author Response:** We thank the reviewer for the suggestion. The description of Fig.14 has been included in the revised manuscript.

The Fig.14 illustrates the class-wise detection performance of the proposed model on the VisDrone dataset, comparing Precision, Recall, mAP@0.5, and mAP@0.5:0.95 across different object categories. It highlights that the model performs consistently well across all classes, achieving the highest scores for larger objects like cars and buses, while maintaining competitive accuracy for small-scale targets such as pedestrians and bicycles. This visualization demonstrates the model’s robustness in handling diverse object sizes and dense aerial scenes.

7. The authors borrowed from YOLO12 and made some improvements, but they didn't compare it to the YOLO12 baseline. The paper's motivation wasn't well explained, and the structure of the improvements wasn't well presented. Overall, the paper lacked innovation or organization.

**Author Response:**

We sincerely thank the reviewer for this insightful feedback. In the revised manuscript, we have included a detailed comparison with the original YOLOv12 baseline to clearly demonstrate the performance gains achieved by our proposed improvements. The motivation section has been rewritten to emphasize the specific limitations of YOLOv12 that our model addresses, such as weak small-object representation and limited attention adaptability. Furthermore, the architectural structure and improvement flow are now reorganized. These revisions strengthen both the clarity and the innovative contribution of the proposed method.
